# The FLIP-FIGNL1 complex regulates the dissociation of RAD51/DMC1 in homologous recombination and replication fork restart

**DOI:** 10.1093/nar/gkad596

**Published:** 2023-07-13

**Authors:** Qianting Zhang, Jiayi Fan, Wei Xu, Huiwen Cao, Cheng Qiu, Yi Xiong, Huacun Zhao, Yong Wang, Jun Huang, Chao Yu

**Affiliations:** The Second Affiliated Hospital, Zhejiang University School of Medicine, Zhejiang University, Hangzhou, China; Zhejiang University-University of Edinburgh Institute (ZJU-UoE Institute), Zhejiang University School of Medicine, Zhejiang University, Haining, China; The MOE Key Laboratory of Biosystems Homeostasis & Protection and Innovation Center for Cell Signaling Network, Life Sciences Institute, Zhejiang University, Hangzhou 310058, Zhejiang, China; College of Life Sciences, Zhejiang University, Hangzhou, China; College of Life Sciences, Zhejiang University, Hangzhou, China; Zhejiang University-University of Edinburgh Institute (ZJU-UoE Institute), Zhejiang University School of Medicine, Zhejiang University, Haining, China; Zhejiang University-University of Edinburgh Institute (ZJU-UoE Institute), Zhejiang University School of Medicine, Zhejiang University, Haining, China; The MOE Key Laboratory of Biosystems Homeostasis & Protection and Innovation Center for Cell Signaling Network, Life Sciences Institute, Zhejiang University, Hangzhou 310058, Zhejiang, China; College of Life Sciences, Zhejiang University, Hangzhou, China; Zhejiang Provincial Key Lab of Geriatrics and Geriatrics Institute of Zhejiang Province, Department of Geriatrics, Zhejiang Hospital, Hangzhou 310030, Zhejiang, China; Zhejiang Provincial Key Laboratory of Cancer Molecular Cell Biology, Life Sciences Institute, Zhejiang University, Hangzhou 310058, Zhejiang, China; College of Life Sciences, Zhejiang University, Hangzhou, China; Assisted Reproduction Unit, Department of Obstetrics and Gynecology, Sir Run Run Shaw Hospital, Zhejiang University, School of Medicine, Hangzhou, China; Key Laboratory of Reproductive Dysfunction Management of Zhejiang Province; Zhejiang Provincial Clinical Research Center for Obstetrics and Gynecology, Zhejiang, China

## Abstract

Recruitment of RAD51 and/or DMC1 recombinases to single-strand DNA is indispensable for homology search and strand invasion in homologous recombination (HR) and for protection of nascent DNA strands at stalled replication forks. Thereafter RAD51/DMC1 dissociate, actively or passively, from these joint molecules upon DNA repair or releasing from replication stress. However, the mechanism that regulates RAD51/DMC1 dissociation and its physiological importance remain elusive. Here, we show that a FLIP-FIGNL1 complex regulates RAD51 and DMC1 dissociation to promote meiotic recombination and replication fork restart in mammals. Mice lacking FLIP are embryonic lethal, while germline-specific deletion of FLIP leads to infertility in both males and females. FLIP-null meiocytes are arrested at a zygotene-like stage with massive RAD51 and DMC1 foci, which frequently co-localize with SHOC1 and TEX11. Furthermore, FLIP interacts with FIGNL1. Depletion of FLIP or FIGNL1 in cell lines destabilizes each other and impairs RAD51 dissociation. Thus, the active dissociation of RAD51/DMC1 by the FLIP-FIGNL1 complex is a crucial step required for HR and replication fork restart, and represents a conserved mechanism in somatic cells and germ cells.

## Graphical Abstract

**Graphical Abstract:**
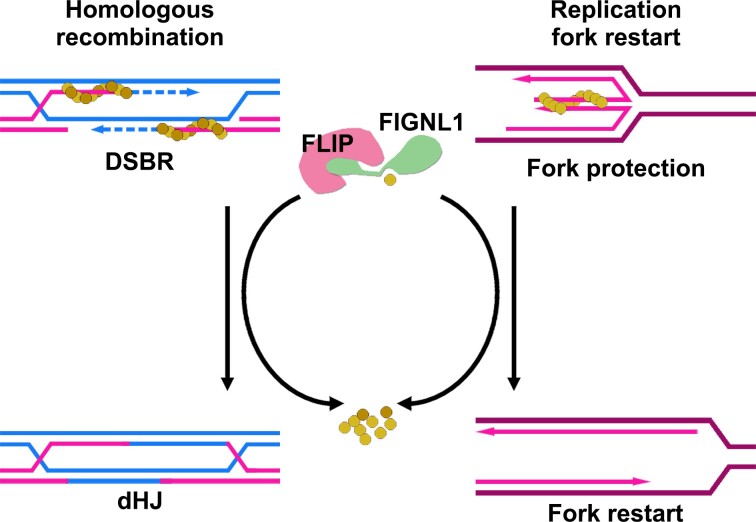

## INTRODUCTION

Homologous recombination (HR) is a high-fidelity repair pathway required for not only DNA double-strand breaks (DSBs) repair and DNA damage tolerance in mitosis, but also formation of crossovers (COs) between homologous chromosomes in meiosis (1,2). Abnormalities in HR efficiency is a common cause of genetic diseases such as Fanconi anaemia-like disorder, cancers, aneuploidy and infertility (3–5). Recombinases RAD51 and its meiosis-specific paralogue, DMC1, play central roles in HR (6,7). Following the formation of either accidentally-occurred DSBs in mitosis or programmed DSBs in meiosis and the subsequent resection, RAD51 and DMC1 are recruited to resected 3′-single strand DNA (3′-ssDNA). The resulting RAD51/DMC1-filaments mediate homology search and strand invasion to form Displacement-loops (D-loops) between DNA molecules, i.e. sister chromatids in mitosis and homologous chromosomes in meiosis (8,9). These nascent joint molecules undergo two pathways. One is the DSB repair (DSBR) pathway involves the processing of D-loops as SEIs (single-end invasions) and dHJs (double Holliday junctions), which are finally resolved as COs and NCOs (non-crossovers) (10,11). The second pathway is the NCO-forming synthesis dependent strand annealing (SDSA) pathway, whereby D-loops are destabilized, and the extended 3′-ssDNA anneals to the complementary strand at the other end of the DSB (2). Many HR- or meiosis-related proteins exert their anti-HR or pro-HR roles at the level of RAD51/DMC1-associated D-loops.

Especially in meiotic recombination, a family of ZMM proteins (Zip1-4, Mer3, Msh4-5 and Spo16 in budding yeast, as well as their mammalian homologs) function in cohort to promote the D-loops being processed through the CO-prone DSBR pathway to guarantee CO formation between homologous chromosomes (12). As with the progression of meiotic recombination, the numbers of RAD51 and DMC1 foci decrease whereas the number of ZMM foci increase, indicating the removal of RAD51/DMC1 (2,13). Similarly, RAD51 is also recruited to stalled replication forks upon replication stress, and mediates replication fork reversal through annealing of two nascent DNA strands (14–16). In this way, the new synthesized leading and lagging strands are protected from endonuclease-mediated degradation and thus, upon releasing from replication stress, RAD51 is removed and replication fork restarts (17). Numerous studies have focused on the mechanisms of how RAD51/DMC1 are charged to the ssDNA in mitosis and meiosis. However, the mechanisms that regulates RAD51/DMC1 dissociation from joint molecules remain elusive. In particular, the physiological importance of RAD51/DMC1 removal in HR and protection of stalled replication forks are largely unknown.

FIGNL1, or Fidgetin-like 1, is a member of the AAA + ATPases and has been identified as a RAD51-binding protein (18,19). FIGNL1 interacts with RAD51 via its internal FRBD (FIGNL1’s RAD51 Binding Domain) and is required for HR. In FIGNL1-depleted cells, the assembly of RAD51-ssDNA in response to DNA damage is not affected, suggesting a post-assembly role of FIGNL1 in regulating RAD51 (18). A recent study suggests a role of FIGNL1 in mediating dissociation of RAD51 from ssDNA (20). FIGNL1 possesses the activity of dismantling ssDNA-bound RAD51 in an ATPase-independent manner. Consistent with these observations, mutants of plant *FIGNL1* homologs, *figl1* in *Arabidopsis thaliana* and *fignl1* in rice, lead to increased numbers of DMC1 or RAD51 foci, as well as COs in meiosis (21,22). Moreover, loss of *figl-1* in *Caenorhabditis elegans* results in infertility due to germ cell depletion (23). Yet the physiological functions of FIGNL1 and its regulatory mechanism in mammalian meiosis is not studied.

FLIP is identified in *A. thaliana* as an interactor of FIGL1 and functions in complex with FIGL1 to regulate meiotic recombination and CO formation (24). Mutation of *flip* in *A. thaliana* phenocopies mutations in *figl1*, resulting in accumulated DMC1/RAD51 foci and increased number of COs (24,25). C1ORF112 is identified as the mammalian homologue of *At*FLIP and therefore termed FLIP (24,26). A genetic screening in mammalian cells identified C1ORF112 as a crucial regulator of DNA interstrand crosslink (ICL) repair and genome stability (27). FLIP is also present as a hint in the anti-FIGNL1 immunoprecipitants in human cell lines, indicating a conserved FLIP-FIGNL1 interaction, or potentially, function in mammals and plants (18,24). The questions of whether FLIP and FIGNL1 function in complex to negatively regulate RAD51/DMC1 assembly, and what is the physiological importance of RAD51/DMC1 removal in meiotic recombination and replication fork restart are not answered.

In this study, we investigated the physiological functions of mammalian FLIP, functioning in complex with FIGNL1, to regulate RAD51/DMC1 dissociation to ensure proper meiotic recombination and replication fork restart. FLIP interacts with the N-terminal 150 amino acids (aa) of FIGNL1. Deletion of FLIP in spermatocytes destabilizes FIGNL1 and leads to RAD51 and DMC1 retention on recombination intermediates, while *FLIP*- or *FIGNL1*-knockout U2OS cells exhibits RAD51 persistence on stalled replication forks. These ectopically-accumulated RAD51/DMC1 in turn arrest meiocytes at a ‘zygotene-like’ stage and U2OS cells to restart the stalled replication forks. Taken together, our results emphasize the physiological importance of the FLIP-FIGNL1 complex as well as active RAD51/DMC1 dissociation to facilitate HR and replication forks restart in mammals.

## MATERIALS AND METHODS

### Mice

**Figure 1.**
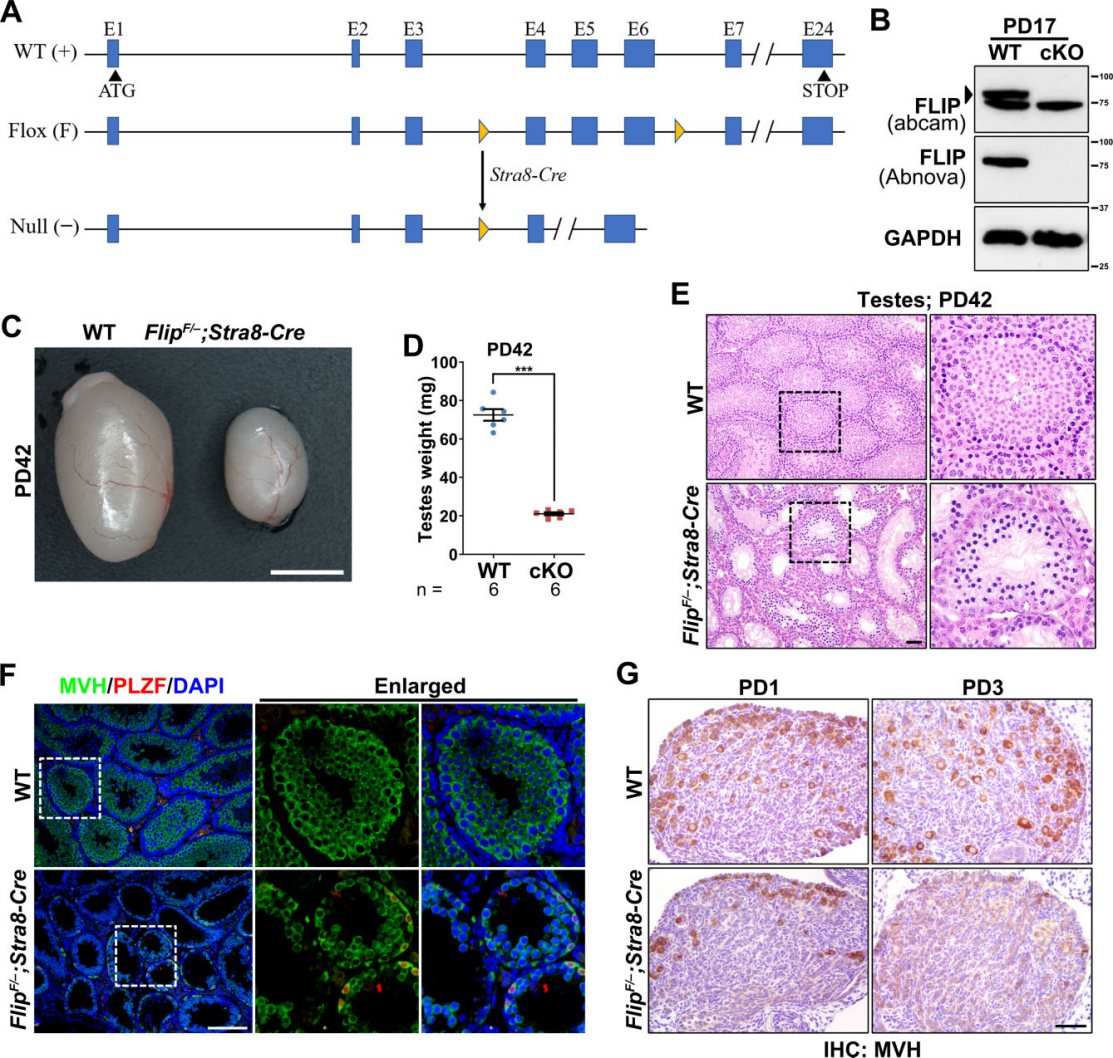
FLIP is required for meiosis in both males and females. (**A**) Schematic diagram showing the gene structure of *Flip*, as well as the strategy to generate the floxed allele (F) and null allele (–) of *Flip*. *Stra8-Cre* is used to delete the genomic sequence between the two loxP sites. The exons, start codon and stop codon are indicated. Yellow triangles indicate the loxP sites. (**B**) Western blotting results showing the knockout efficiency of FLIP in postnatal day 17 (PD17) testes. The size of protein markers is indicated. Black triangle indicates the specific band of certain antibody. cKO, conditional knockout, i.e. *Flip^F/–^;Stra8-Cre*. (**C**) A representative image showing the size of testes derived from WT and *Flip^F/–^;Stra8-Cre* males at PD42. Scale bar, 2 mm. (**D**) Weights of WT and *Flip^F/–^;Stra8-Cre* testes at PD42. Numbers of testes analyzed (*n*) are indicated. Error bars indicate S.E.M. ***P < 0.001 by two-tailed Student's *t* test. (**E**) Hematoxylin & eosin (H&E) staining results of WT and *Flip^F/–^;Stra8-Cre* testes at PD42. The regions within dashed lines are enlarged on the right. Scale bar, 50 μm. (**F**) Immunofluorescent (IF) staining of MVH (green) and PLZF (red) in WT and *Flip^F/–^;Stra8-Cre* testes sections at PD42. The regions within dashed lines are enlarged on the right. Scale bar, 100 μm. (**G**) Immunohistochemistry (IHC) staining of MVH in ovaries sections derived from WT and *Flip^F/–^;Stra8-Cre* females at PD1 and PD3. Scale bar, 50 μm.

Mice (C57BL/6) carrying the *Flip* floxed allele (*Flip^F^*) were generated via CRISPR-Cas9 technique in GemPharmatech Co., Ltd. *Stra8-Cre* mouse stain gifted from Dr Ming-Han Tong was previously reported (28). *Flip^F/F^* mice were crossed with *Stra8-Cre* resulting in germ cell-specific *Flip* knockout mice, i.e. *Flip^F/^*^–^*;Stra8-Cre*. The schematic in Figure 1A illustrates *Flip* gene structures in wildtype (WT), Floxed (Flox) and knockout (Null) alleles as well as the targeting strategies. All mice were housed following the SPF protocol in a steady environment (12 light/12 dark cycle, temperature of 22°C with 50–70% humidity, and adequate sterilized feed and water). Animal care and experimental procedures were supervised by Laboratory Animal Welfare of Ethics Committee of Zhejiang University (approval #ZJU20210226). The genotyping primers are listed in Supplementary Table S1.

### Embryo assay

*Flip^+/–^* males were crossed to *Flip^+/–^* females, and vaginal plugs were checked in the next morning, which corresponds to embryonic day 0.5 (E 0.5). The plugged females were sacrificed at E10.5 and E13.5 with their uteruses completely dissected to examine the quantities and sizes of embryos. The embryos were subsequently dissected out of the uterus to observe their external morphology under a stereo microscope (Nikon).

### Western blot analysis

Testes were lysed thoroughly in SDS sample buffer (25 mM Tris–HCl [pH 6.8], 2% SDS, 10% glycerol, 5% β-mercaptoethonol and 0.01% bromophenol blue) by sonication and boiled at 95°C for 5 min. Protein samples were prepared and separated by SDS-PAGE technique, transferred to PVDF membranes (Immobilon^®^-P, #IPVH00010), followed by sequential incubation in blocking buffer (30 min), primary antibody dilution (1 h), wash buffer (TBS with 0.05% Tween-20), HRP-conjugated secondary antibody (Jackson ImmunoResearch) dilution (1 h) and wash buffer. The chemiluminescent signals were detected with SuperSignal™ West Pico PLUS Kit (Thermo Fisher, #34577). The primary antibodies used and dilution ratios are listed in Supplementary Table S2.

### Histological analyses

Testes, epididymides and ovaries were dissected and fixed in PBS buffered 3.7% formaldehyde, subsequently dehydrated with an ethanol gradient and xylene, and finally embedded in paraffin for preparation of 5-μm thick sections. For H&E staining, paraffin-embedded sections were deparaffinized with xylene, rehydrated with an alcohol gradient, rinsed with water and stained with hematoxylin and eosin respectively (30–60 s each). The testes could also be fixed in Bouin's solution for better histology of seminiferous tubules. For immunohistochemistry (IHC) staining, after deparaffinization, rehydration, and peroxidase blocking in 3% hydrogen peroxide for 10 min, antigen unmasking was performed on slides by boiling in 10 mM Sodium Citrate Buffer (pH 6.0) at 95°C for 15 min and cooling on bench to room temperature. The slides were successively incubated with blocking buffer (30 min), primary antibody dilutions (1 h) and biotinylated secondary antibody dilutions (30 min), labeled with Vectastain ABC kit (Vector Laboratories) and finally stained with DAB peroxidase substrate kit (Vector Laboratories). The primary antibodies involved are listed in Supplementary Table S2.

### Immunofluorescent staining and imaging

Paraffin-embedded sections were deparaffinized and rehydrated similarly as IHC assay. The slides were then blocked with 1% BSA in PBS with 0.1% Tween-20 (30 min), and sequentially incubated with diluted primary antibodies (1 h), Fluorophore (FITC or CY3)-conjugated secondary antibodies (Jackson ImmunoResearch, 30 min) and 5 μg/ml DAPI (10 min). After thoroughly rinse in PBS, the slides were mounted with 80% glycerol. The signals were examined under Upright Fluorescence Microscope (Nikon) and images were collected with Confocal Laser Scanning Microscope (Olympus, FV3000) or Nikon Eclipse 80i Fluorescence Microscope for quantification. The antibodies applied were listed in Supplementary Table S2.

### TUNEL assay

The experiments were conducted following the protocol of TUNEL BrightGreen Apoptosis Detection Kit (Vazyme, #A112-01). In brief, paraffin-embedded sections were deparaffinized plus rehydrated, followed by successive steps of Proteinase K digestion, equilibration, FITC-12-dUTP labeling, rinse in PBS, contra-staining with DAPI and mounting in glycerol. The imaging process is in the same way as IF assay. Finally, the FITC-positive cells in seminiferous tubules were calculated for statistical analysis.

### Nuclear surface spreading

The experiments were performed as previously described (29). Briefly, seminiferous tubules derived from juvenile and adult testes were immersed in Hypotonic Extraction Buffer (30 mM Tris–HCl [pH8.2], 50 mM sucrose, 17 mM trisodium citrate dehydrate, 5 mM EDTA and 0.5mM DTT) for 30 min and thoroughly smashed with sharp forceps in 100 mM Sucrose Solution (pH 8.2) to disperse testicular cells. An aliquot of 20-μ cell suspension was added on the surface of a slide containing Fixative Buffer (1% paraformaldehyde and 0.15% Triton X-100, pH 9.2). The slides were kept in a humidify box for 2 h to become air dried and subjected to immunofluorescent staining. Fluorescent foci of recombination-associated proteins on chromosomes were examined and quantified in around 20 spermatocytes at each stage of meiotic prophase I as well as metaphase I of each experimental group.

### Structure prediction and molecular dynamics simulation

The structures of the FLIP-FIGNL1 complex were predicted using AlphaFold multimer version 2.2 which was installed locally with default pipeline (30). The complex was placed in a 12.2 nm cubic box and solvated with TIP3P water molecules containing Na^+^ and Cl^−^ ions at 0.15 M. A minimal model of FLIP (aa. 1–150) and FIGNL1 (aa. 258–840) was constructed to reduce computational cost, resulting in ∼238 000 atoms in total. The Amber ff99SB-disp force field was used for all simulations (31). Temperature and pressure were kept constant at 300 K and 1.0 bar using the V-rescale thermostat and Parrinello-Rahman barostat, respectively. Neighbor searching was performed every 5 steps. The PME algorithm was used for electrostatic interactions with a single cutoff of 1.0 nm for both PME and van der Waals interactions. A reciprocal grid of 80 × 80 × 80 cells was used with fourth-order B-spline interpolation. The hydrogen mass repartitioning technique was used with a single linear constraint solver (LINCS) iteration, allowing simulations to be performed with an integration time step of 4 fs. MD simulations were performed using GROMACS 2021.5 (32,33). Protein structures were visualized with PyMOL 2.5.

### Cloning of cDNA

cDNA encoding *Flip* and *Fignl1*, *Rad51*, *Dmc1* and *Swsap1* were amplified from mouse testes cDNA. The deletion mutants FLIP-ΔN230, FLIP-ΔN350, FLIP-DUF and FIGNL1-ΔFRBD, FIGNL1-N, FIGNL1-ΔN370, FIGNL1-ΔN300, FIGNL1-ΔN150 were generated by fusion PCR. All the above cDNA fragments were subcloned into modified pRK5 (Genentech) vectors, pRK5-FLAG, pRK5-HA, pRK5-GFP or pRK5-FLAG-GFP for expression of N-terminal tagged recombinant proteins. Full-length cDNA clones encoding human FLIP and FIGNL1 were cloned into pDONOR201 gateway entry vector (Invitrogen) and then recombined into SFB-tagged destination vector. Plasmids were transfected by polyethyleneimine. sgRNA oligos targeting human *FLIP* and *FIGNL1* were subcloned into pSpCas9(BB)-2A-puro vector by annealing and ligation. The successful cloning was verified by DNA sequencing.

### Cell lines and immunoprecipitation

293T and U2OS cells were cultured in DMEM (Sigma-Aldrich, #D6546) medium supplemented with 10% fetal bovine serum at 37°C in 5% CO_2_ air atmosphere. Cells were transiently transfected with plasmids carrying FLAG, HA or GFP-tagged proteins using Lipofectamine 2000 (Invitrogen, #11668019), and harvested 24 h after transfection. Whole cell lysates were prepared with nuclear lysis buffer (25 mM Tris–HCl [pH 7.5], 300 mM NaCl, 1% Triton X-100, 1 mM DTT, 1 mM EDTA, protease inhibitors added before use), followed by sonication, and then precipitated with antibody-conjugated agarose beads or antibodies plus Protein A agarose beads (Pierce™, #20333) as indicated in relevant figures. The lysates-beads mixtures were incubated in a rotating shaker at 4°C for 4 h and washed thoroughly in nuclear lysis buffer. The whole cell lysates and immunoprecipitated samples were boiled in SDS sample buffer at 95°C for 10 min and subjected for western blotting analysis. The antibody-conjugated agarose beads used were listed in Supplementary Table S2.

### Generation of knockout U2OS cell lines

U2OS cells were transiently transfected with pSpCas9(BB)-2A-puro-sgRNA plasmids with lipofectamine for 6 h; and 24 h later, treated with 0.5 μg/ml puromycin for 40 h. The retained cells were diluted into 96-well plates for culturing single colonies. As the colonies were grown up and transferred to 24-well plates, *FLIP* or *FIGNL1* depletion were examined by cell harvest, genomic DNA purification, PCR amplification of sgRNA-targeted fragments, DNA sequencing and sequence analysis. The colonies with both alleles carrying the gene-knockout mutations were expanded and reserved for further investigations. The sgRNAs designed for *FLIP* or *FIGNL1* depletion were indicated in Supplementary Figure S8. The primers used for *FLIP* or *FIGNL1* genotyping in human cells were listed in Supplementary Table S1.

### siRNA transfection

siRNAs against FLIP and FIGNL1 were transfected by RNAiMAX (Thermo Fisher Scientific, #13778075) according to the manufacturer's directions. Transfection was repeated twice with an interval of 24 h to achieve maximal RNAi effect. The siRNA sequences used for *FLIP* or *FIGNL1* knockdown in human cells were listed in Supplementary Table S3.

### Tandem affinity purification

Twenty 10-cm dishes of HEK293T stably expressing SFB-tagged wild-type FLIP (human) or FIGNL1 (human) were cultured. After 24 h, the cells were treated with 4 mM HU for 3 h and then lysed in NETN buffer (20 mM Tris–HCl [pH 8.0], 100 mM NaCl, 1 mM EDTA, and 0.5% Nonidet *P*-40) supplemented with 500 U/ml benzonase (Novagen) and protease inhibitors (1 μg/ml aprotinin and leupeptin) for 30 min at 4°C. The resulting crude lysates were centrifuged at 14 000 g for 10 min, and the supernatants were collected and incubated with 200 μl streptavidin-sepharose beads (Amersham Biosciences) for 3 h at 4°C.The beads were then washed three times with NETN buffer, and the bound proteins were eluted with washing buffer containing 1 mg/ml biotin (Sigma-Aldrich) for 1 h at 4°C. The elutes were then incubated with S‐protein beads (Novagen) for 2 h at 4°C. The beads were again washed three times with NETN buffer and subjected to SDS-PAGE. Protein bands were excised and digested, and the peptides were analyzed by mass spectrometry (34).

### Proximity ligation assay

U2OS cells were pulse-labeled with 10 μM EdU for 15 min. Subsequently, the cells were either left untreated or treated with 4 mM HU for 3 h before they were washed with PBS. Next, the cells were permeabilized with 0.5% Triton X-100 for 5 min, fixed with a solution of 3% formaldehyde and 2% sucrose in PBS at room temperature for 10 min, and blocked with 3% BSA in PBS for 30 min. After washing with PBS, the cells were subjected to Click-iT reaction to attach biotin to EdU, and then incubated overnight at 4°C with the appropriate primary antibodies. The proximity ligation assay was performed using the Duolink In Situ Red Starter kit (Sigma-Aldrich) according to the manufacturer's instructions. Images were acquired using a Nikon Eclipse 80i Fluorescence Microscope equipped with a Plan Fluor 60× oil objective lens (NA 0.5–1.25; Nikon) and a camera (CoolSNAP HQ2; Photometrics), and analyzed with NIS-Elements basic research imaging software (Nikon).

### Recombinant protein purification

Plasmids encoding FLIP, FIGNL1 and RAD51 proteins fused with GST or MBP tags were generated by subcloning PCR fragments into pCold-GST or pCold-MBP vectors. The constructed plasmids were then transformed into BL21 (DE3) competent cells. Cells were cultured in LB broth with appropriate antibiotics at 37°C until reaching the logarithmic growth phase. Protein expression was induced by adding 0.4 mM isopropyl β-d-1-thiogalactopyranoside and incubating at 16°C for 16 hours. After harvesting the cells, they were lysed using sonication in lysis buffer (20 mM Tris–HCl pH 8.0, 300 mM NaCl, 1% Triton X-100 and 1 μg/ml each of leupeptin and aprotinin). The lysate was clarified by centrifugation, and the resulting supernatant was incubated with glutathione sepharose 4B (GE Healthcare) or amylose resin (BioLab) in lysis buffer at 4°C for 8 h. Following incubation, the beads were washed three times with washing buffer (20 mM Tris–HCl pH 8.0, 500 mM NaCl, 0.5% NP-40, and 1 μg/ml each of leupeptin and aprotinin). The bound proteins were then eluted from the beads using washing buffer containing 20 mM glutathione or 10 mM maltose for subsequent *in vitro* GST pull-down assays.

### *In vitro* GST pull-down assay

For the GST pull-down assay, 0.05 μg of bacterially purified GST or GST-FLIP was combined with Glutathione agarose beads and 0.1 μg of bacterially expressed MBP, MBP-FIGNL1, or MBP-RAD51 in a total volume of 800 μl of NETN buffer (20 mM Tris HCl [pH 8.0], 100 mM NaCl, 1 mM EDTA and 0.5% Nonidet *P*-40). The mixture was incubated at 4°C for 30 min to allow protein binding. After incubation, the beads were washed three times with NETN buffer to remove unbound proteins. Then, the beads were boiled in 2× SDS-PAGE loading buffer, and the resolved proteins were subjected to immunoblot analyses using the specified antibodies.

### DNA fiber analysis

U2OS cells were incubated with 50 μM IdU for 20 min, followed by exposure to 4 mM HU for 3 h. After removing HU, the cells were incubated with 250 μM CldU (Sigma-Aldrich, #C6891) for 20 min. The labeled cells were trypsinized and resuspended in ice-cold PBS at a concentration of 1 × 10^6^ cells per milliliter. A total of 2.5 μl of cell resuspension were spotted onto a pre-cleaned glass slide and lysed with 7.5 μl of spreading buffer (0.5% SDS in 200 mM Tris–HCl [pH 7.4], 50 mM EDTA). After 5 min, the slides were tilted at 15° relative to horizontal and the resulting DNA spreads were air-dried, fixed in a solution of 3:1 methanol/acetic acid for 20 min at –20°C, and then denatured with 3 M HCl overnight at 4°C. Following washing with PBS, the slides were blocked with 1% BSA in PBS for 30 min and incubated with anti-IdU/BrdU and anti-CldU/BrdU antibodies to detect IdU and CldU, respectively. After a 3 h incubation, the slides were washed with PBS and stained with Rhodamine-conjugated goat anti-mouse IgG (Jackson ImmunoResearch) and Alexan Fluor 488 Donkey anti Rat IgG (Life technologies) for 2 h at room temperature in the dark. Images were acquired using a Nikon Eclipse 80i Fluorescence Microscope equipped with a Plan Fluor 60× oil objective lens (NA 0.5–1.25; Nikon) and a camera (CoolSNAP HQ2; Photometrics), and analyzed with NIS-Elements basic research imaging software (Nikon). The DNA tract lengths were measured using ImageJ, and the pixel length values were converted into micrometers using the scale bars created by the microscope. In each experiment, a minimum of 100 individual tracks were measured. The antibodies used were listed in Supplementary Table S2.

### Homologous recombination assay

0.5  ×  10^6^ U2OS DR-GFP cells were seeded and subsequently electroporated with 3 μg of I-SceI expression plasmid (pCBASce) after 24 h. Cells were harvested 48 h post pCBASce electroporation and subjected to flow cytometry analysis to measure GFP expression, which reflects the efficiency of homologous recombination (HR) in repairing the I-SceI-induced double-stranded break. The percentages of GFP-positive cells were determined and the means were obtained from three independent experiments.

### Statistics

All the experiments have been repeated at least three times with independent biological samples. Dot plots and bar graphs were created with means ± S.E.M. The differences between group means were calculated by either two-tailed Student's *t* test or one-way ANOVA. The level of statistical significance is expressed as *P* < 0.05 (*), *P* < 0.01 (**) or *P* < 0.001 (***).

## RESULTS

### FLIP is essential for embryogenesis

Mouse *Flip*, or *BC055324* (gene ID: 381306), is the homologue of human *FLIP* (gene ID: 55732) and consists of 24 exons encoding a protein of 903 aa in length. To investigate the functions of FLIP in mice, we constructed a *Flip* conditional allele. In this allele, the left loxP site is inserted in the intron between exon 3 and exon 4, while the right loxP site is inserted in the intron between exon 6 and exon 7 (Figure 1A and Supplementary Figure S1A). *Stra8-Cre* knockin mouse strain, which expresses Cre recombinases exclusively in meiotic germ cells, was used to achieve both conventional and conditional knockout of *Flip* in this study (28).

Heterozygous *Flip^+/–^* mice were obtained by crossing *Flip^F/+^;Stra8-Cre* males to WT females. However, *Flip^+/–^* male to *Flip^+/–^* female breeding results in no homozygous *Flip^–/–^* mice (0/56 from 9 l), indicating that *Flip^–/–^* embryos are not viable. We then checked the vaginal plugs of *Flip^+/–^* to *Flip^+/–^* breeding and dissected pregnant females at the stages of embryonic day 10.5 (E10.5) and E13.5. Degenerated deciduae were frequently seen in these uteruses (Supplementary Figure S1B, C). In E10.5 uteruses, 4 out of 22 deciduae appear smaller with placentas and remaining embryos (Supplementary Figure S1D). And in E13.5 uteruses, 9 out of 30 deciduae were degenerated (Supplementary Figure S1E). These results suggest that conventional deletion of FLIP leads to embryonic lethality.

### FLIP-deletion leads to male and female infertility

On the other hand, breeding of *Flip^F/+^;Stra8-Cre* males with *Flip^F/F^* females produces the conditional knockout *Flip^F/–^;Stra8-Cre* mice (or *Flip* cKO). The knockout efficiency in *Flip^F/–^;Stra8-Cre* testes was confirmed by Western blotting using two FLIP antibodies (Figure 1B). Interestingly, both *Flip^F/–^;Stra8-Cre* males and females are infertile. Testes derived from *Flip* cKO males at PD42 are significantly smaller than WT controls (Figure 1C-D). The weight of *Flip* cKO testes is 21.05 ± 0.80 mg (compared to 72.48 ± 3.01 mg in WT, *P* = 1.37 × 10^−8^). This testes size is close to that of *Spo11*, *Dmc1*, *Shoc1* or *Spo16-*knockout males, in which spermatogenesis is arrested at meiotic prophase I, but smaller than the metaphase I-arrested *Psma8* knockout testes (35–39). Consequently, hematoxylin and eosin (H&E) staining of these testes shows that spermatogenesis is arrested at meiotic prophase I (Figure 1E). MVH (mammalian vasa homologue, a marker of germ cells) and PLZF (promyelocytic leukemia zinc finger, a marker of undifferentiated and differentiating spermatogonia) were further stained in WT and *Flip* cKO testes sections (40,41). While WT seminiferous tubules are full of MVH-positive cells including spermatogonia, spermatocytes and round/elongated spermatids, the seminiferous tubules in *Flip* cKO testes are smaller with large cavities and no round or elongated spermatids were found (Figure 1E, F). WT1, or Wilm's tumor 1, is expressed in the somatic Sertoli cells in testes (42). FLIP-deletion has compromised effects on spermatogonia, since there is no significant difference in the ratio of cells positive for PLZF to cells positive for WT1 in WT and *Flip* cKO testes (Supplementary Figure S2A, B). TUNEL (TdT-mediated dUTP Nick-End Labeling) assay results show massive apoptotic cells in *Flip* cKO testes (Supplementary Figure S2C, D). While WT epididymides are full of mature spermatozoa at PD42, epididymides derived from *Flip* cKO males are always empty (Supplementary Figure S2E).

Similarly, ovaries derived from *Flip^F/–^;Stra8-Cre* females are smaller (Supplementary Figure S3A). Especially in *Flip* cKO females beyond the age of PD25, ovaries are almost missing and hard to be dissected from the oviducts (Supplementary Figure S3A). *Flip* cKO females have fewer oocytes at PD1 and PD3, as evidenced by both H&E staining and MVH immunohistochemical staining (Figure 1G and Supplementary Figure S3B, C). Moreover, no oocytes were observed in PD10 *Flip* cKO ovaries, suggesting a phenotype of premature ovarian failure (Supplementary Figure S3D).

### Impaired meiotic prophase I progression in FLIP-null spermatocytes

**Figure 2.**
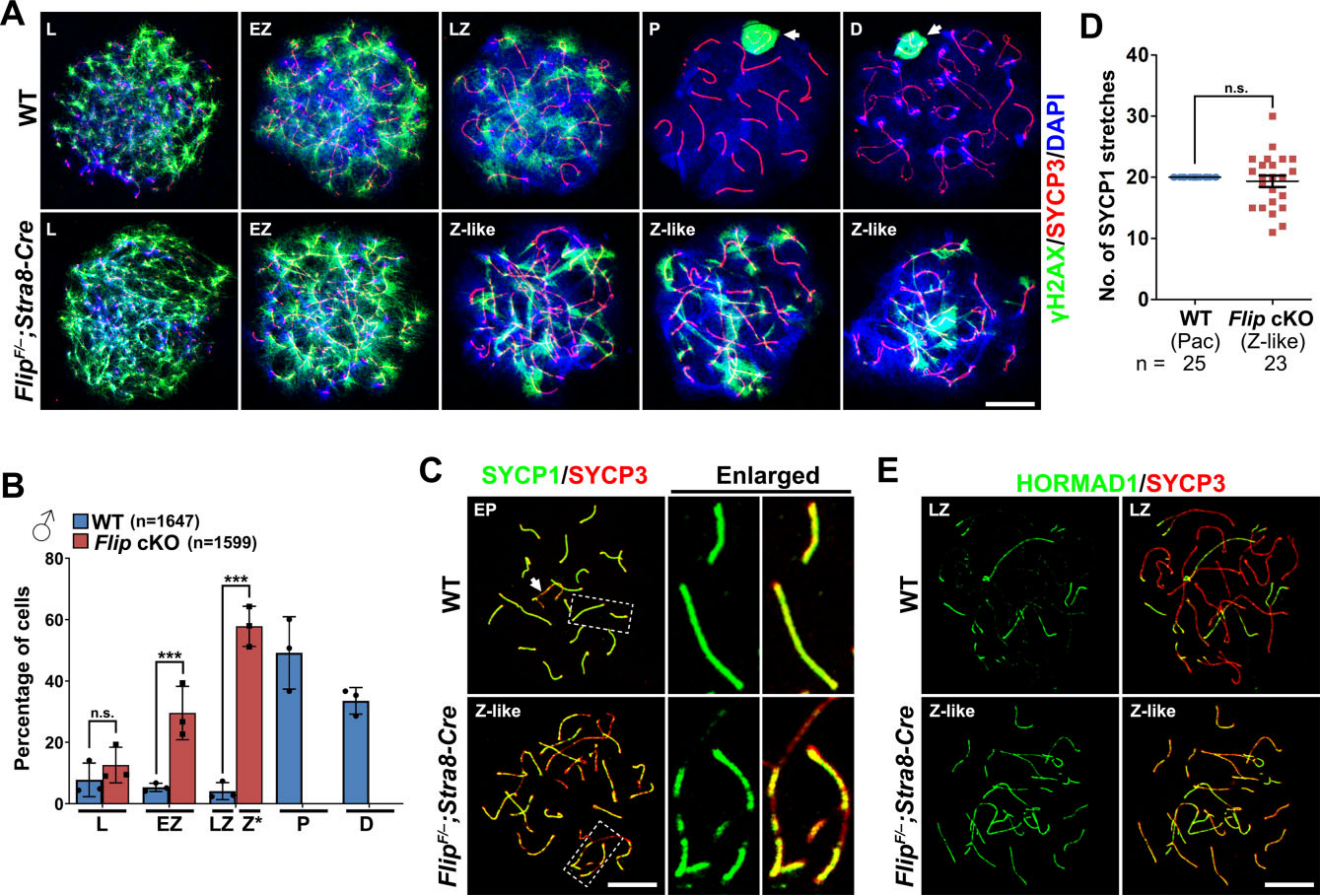
Defective meiotic prophase I progression in FLIP-deleted spermatocytes. (**A**) γH2AX (green) and SYCP3 (red) were co-stained on the nuclear surface spreads prepared with WT and *Flip^F/–^;Stra8-Cre* testes at PD21 to exhibit the status of DNA double strand breaks (DSB) in meiotic prophase I. L, leptotene; EZ, early zygotene; LZ, late zygotene; P, pachytene; D, diplotene; Z-like, zygotene-like. Scale bar, 10 μm. (**B**) Meiotic prophase I progression in WT and *Flip^F/–^;Stra8-Cre* testes. The percentages of spermatocytes at each developmental stage during meiotic prophase I were calculated. Error bars indicate S.E.M. The numbers of spermatocytes analyzed (*n*) are indicated. Z*, zygotene-like. n.s., no significance; ***, *P* < 0.001 (two-tailed Student's *t* tests). (**C**) IF staining of SYCP1 (green) and SYCP3 (red) on the nuclear surface spreads of spermatocytes derived from WT and *Flip^F/–^;Stra8-Cre* males at PD21. The regions within dashed lines are enlarged on the right. Scale bar, 10 μm. (**D**) Quantification of SYCP1 stretches in WT spermatocytes at the pachytene stage and *Flip^F/–^;Stra8-Cre* spermatocytes at the zygotene-like stage. *n* indicates the number of cells analyzed. n.s., no significane (two-tailed Student's *t* test). (**E**) Co-staining of unsynapsed chromosome marker HORMAD1 (green) and SYCP3 (red) on the nuclear surface spreads of WT and *Flip^F/–^;Stra8-Cre* spermatocytes. Scale bar, 10 μm.

To elucidate the reason why FLIP-deletion leads to infertility, we set out to investigate meiotic prophase I progression in male testes. γH2AX is a DSBs marker reflecting the status of generation and repair of meiotic DSBs (13). Therefore, in WT spermatocytes, γH2AX signals are detected in the whole nuclear at leptotene and early-pachytene stages, and retract to the unrepaired regions at late-zygotene, pachytene and diplotene stages (Figure 2A). The unrepaired region in pachynema and diplonema is the sex body composed of mainly the XY chromosomes. The patterns of γH2AX signal are comparable in leptonema and early-zygonema *Flip^F/–^;Stra8-Cre* spermatocytes (Figure 2A), suggesting that the generation of meiotic DSBs are not dramatically affected by FLIP deletion. However, pachynema and diplonema with γH2AX signals detected only on sex bodies are not found in *Flip* cKO testes (Figure 2B and Supplementary Figure S4A). Instead, around 60% FLIP-null spermatocytes are arrested at a zygotene-like stage, which is similar to the late-zygonema in WT testes (Figure 2A, B).

Synapsis is another hallmark of meiotic prophase I progression. SYCP1 is the central elements of the synaptonemal complex and therefore indicates the synapsed regions on homologous chromosomes, while HORMAD1 localizes to the unsynapsed chromosome axes (43,44). In WT spermatocytes at the early-pachytene stage, SYCP1 stretches extend to the full length on each pair of homologous chromosomes except for the XY chromosome pair (Figure 2C). However, even in the most advanced zygonema-like spermatocytes null for FLIP, SYCP1 stretches do not elongate to the full length, remaining short in morphology and sometimes increasing in numbers (Figure 2C, D). Incomplete synapsis is also seen in *Flip^F/–^;Stra8-Cre* testes sections stained for SYCP1 and SYCP3, and in FLIP-null spermatocytes stained for HORMAD1 and SYCP3 (Figure 2E and Supplementary Figure S4B). Interestingly, in WT spermatocytes, HORMAD1 is removed from the synapsed chromosome regions, whereas in FLIP-deleted spermatocytes, HORMAD1 signal is detected on the paired chromosomes, although in a lower intensity than on the unpaired regions (Figure 2E). Taken together, these results suggest that FLIP-deleted spermatocytes are arrested at a zygotene-like stage and cannot complete meiotic prophase I.

### FLIP regulates RAD51/DMC1 dissociation in meiocytes

**Figure 3.**
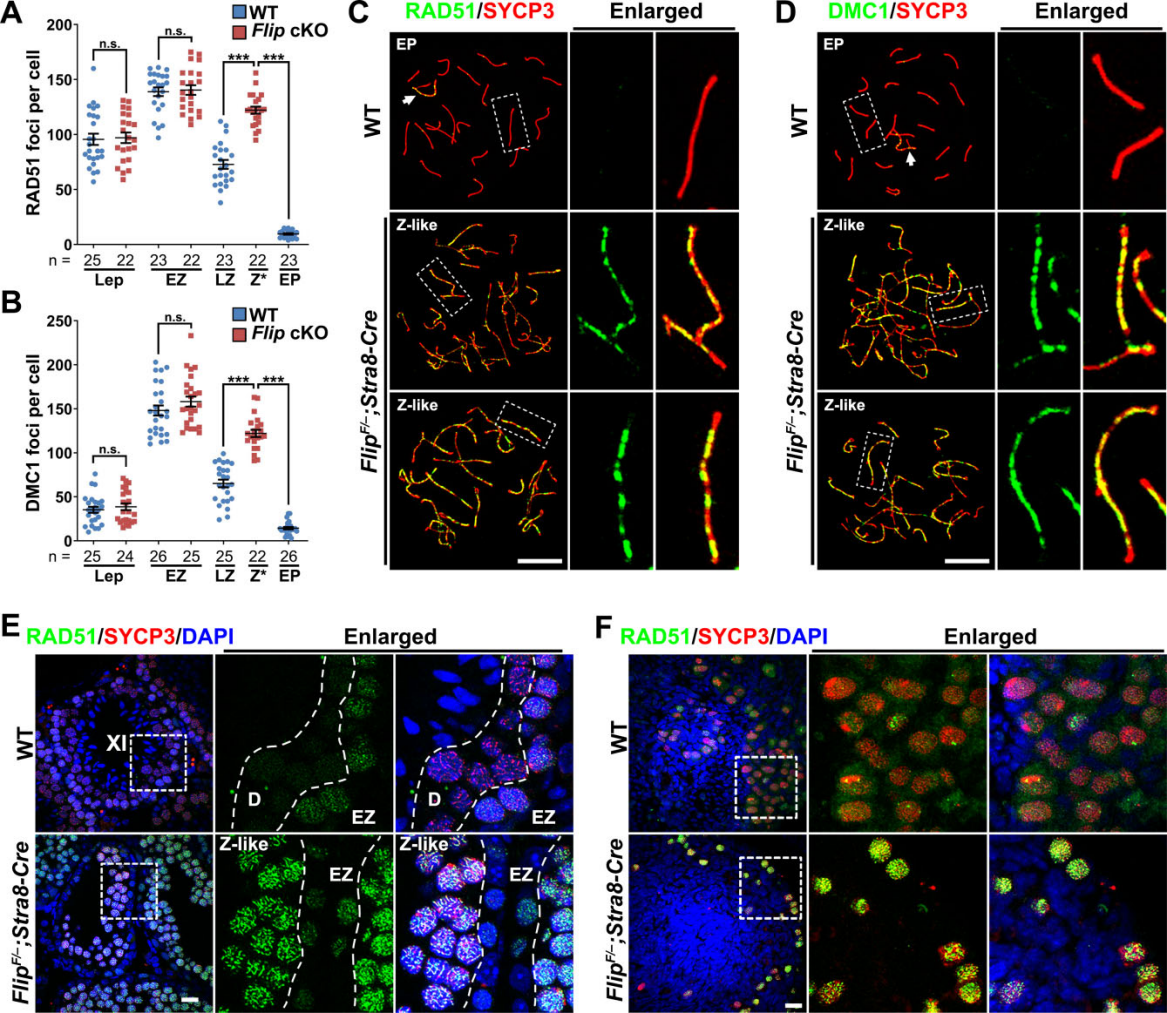
Retention of RAD51 and DMC1 in FLIP-deleted meiocytes. (A, B) Quantification of RAD51 (**A**) and DMC1 (**B**) foci in WT and *Flip^F/–^;Stra8-Cre* spermatocytes at indicated stages. Lep, leptotene; Z*, zygotene-like. n indicates the numbers of cells analyzed. Error bars indicate S.E.M. n.s., no significance; ***P < 0.001 (two-tailed Student's *t* tests). (C, D) Representative images of RAD51 (**C**) and DMC1 (**D**) (green) co-stained with SYCP3 (red) on the nuclear surface spreads of WT pachynema and *Flip^F/–^;Stra8-Cre* spermatocytes at zygotene-like stage. The regions within dashed lines are enlarged on the right. Arrows indicate the sex bodies. Scale bars, 10 μm. (E, F) Co-staining of RAD51 (green) and SYCP3 (red) on testes section at PD42 (**E**) and ovaries sections at PD1 (**F**). The regions within dashed lines are enlarged on the right. The stages of spermatocytes are indicated. XI, a representative seminiferous tubule at stage XI. Scale bars, 20 μm.

We then dissected the processes of meiotic recombination in WT and FLIP-deleted spermatocytes. Recombinases RAD51 and DMC1 are markers of early recombination intermediates in meiosis, since they are recruited to the sites of resected 3′-ssDNA and somehow removed upon dHJ formation (6). Accordingly, in WT spermatocytes, the numbers of RAD51 and DMC1 foci on chromosomes arise in leptonema, increase in early-zygonema and decrease in late-zygonema and early-pachynema (Figure 3A, B and Supplementary Figure S5A, B). Especially in WT spermatocytes at early-pachytene stage, RAD51 and DMC1 foci are restricted mainly to the XY chromosomes (Figure 3C, D). RAD51 foci and DMC1 foci at leptotene and early-zygotene stages are comparable between WT and *Flip* cKO spermatocytes (Figure 3A, B and Supplementary Figure S5A, B). Strikingly, persistent RAD51 foci and DMC1 foci are found in FLIP-deleted spermatocytes at the zygotene-like stage (Figure 3A, D and Supplementary Figure S5A, B). Extensive RAD51 foci and DMC1 foci are detected on both unsynapsed regions and synapsed regions. Compared to a 48% and a 93% reduction of RAD51 foci in WT spermatocytes at the early-zygotene to late-zygotene transition and the early-zygotene to early-pachytene transition, the number of RAD51 foci only decreases at a degree of 13% at the transition from early-zygotene stage to zygotene-like stage (Figure 3, A and C). Similarly, the number of DMC1 foci exhibits a 23% decrease in FLIP-null spermatocytes during the early-zygotene to zygotene-like transition, which is not as dramatically decreased as in WT spermatocytes (Figure 3B and D). As a result, in FLIP-deleted spermatocytes at the zygotene-like stage, the numbers of RAD51 foci and DMC1 foci are significantly more than WT spermatocytes at late-zygotene and early-pachytene stages, suggesting insufficient RAD51/DMC1 removal during meiotic recombination.

Even though, the numbers of RAD51 and DMC1 foci in *Flip* cKO spermatocytes at the zygotene-like stage might have been underestimated, since these foci are often too close with each other to be distinguished on the condensed chromosome axes (Figure 3C, D). Therefore, RAD51 and DMC1 signals were further detected on testes sections. As shown in Figure 3E and Supplementary Figure S5C, the intensity of RAD51 and DMC1 signals in *Flip^F/–^;Stra8-Cre* testes is stronger than in WT testes at comparable stages. Moreover, the defects in RAD51 and DMC1 removal are also apparent in meiotic female primordial germ cells (PGCs) at PD1 (Figure 3F and Supplementary Figure S5D). While WT PGCs are at diplotene stage with no or weak RAD51/DMC1 signals, FLIP-depleted PGCs are RAD51- and DMC1-positive throughout the nucleus.

### Stalled meiotic recombination in FLIP-deleted spermatocytes

**Figure 4.**
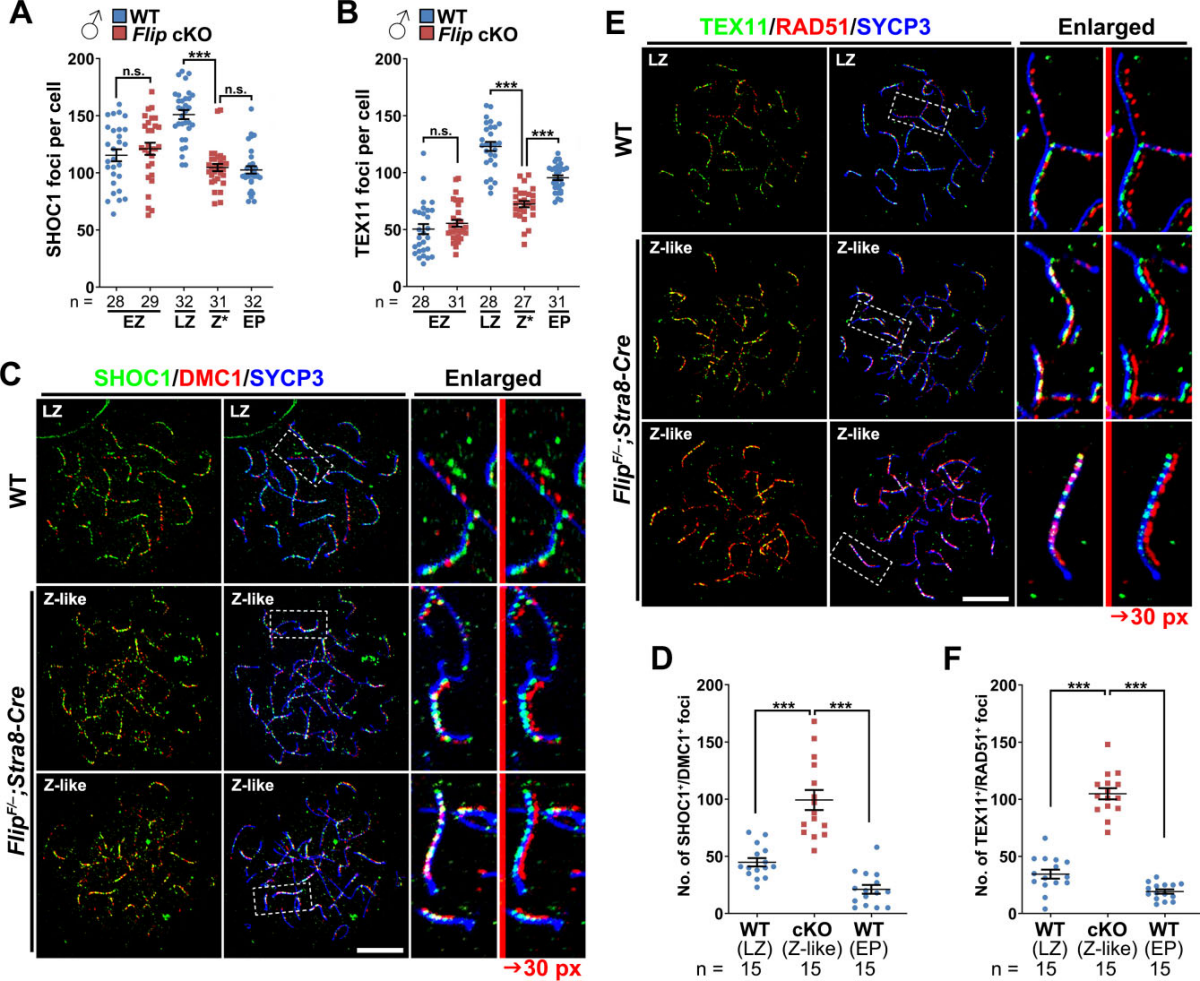
Ectopic co-localization of RAD51/DMC1 and ZMM proteins in FLIP-deleted spermatocytes. (A, B) Quantification of SHOC1 (**A**) and TEX11 foci (**B**) on the nuclear surface spreads prepared with WT and *Flip^F/–^;Stra8-Cre* testes at PD21. Error bars indicate S.E.M. *n* indicates the numbers of cells analyzed. n.s., no significance; ***P < 0.001 (two-tailed Student's *t* tests). (**C**) Triple staining of SHOC1 (green), DMC1 (red) and SYCP3 (blue) on WT and *Flip^F/–^;Stra8-Cre* nuclear surface spreads. The regions within dashed lines are enlarged on the right, and the red signals are further shifted rightwards for 30 pixels to present green-red co-localization. Scale bar, 10 μm. (**D**) Quantification of the SHOC1 and DMC1 co-localized foci in WT late-zygonema and early-pachynema, as well as in FLIP-deleted spermatocytes at the zygote-like stage. Error bars indicate S.E.M. *n* indicates the numbers of cells analyzed. ***P < 0.001 (two-tailed Student's *t* tests). (E, F) Detection (**E**) and quantification (**F**) of TEX11 (green) and RAD51 (red) co-localization on WT and *Flip^F/–^;Stra8-Cre* nuclear surface spreads.

Upon RAD51/DMC1-mediated D-loop formation between homologous chromosomes, a group of ZMM proteins are recruited to these sites for further meiotic recombination process, i.e. formation of SEI and dHJ (45). SHOC1 is one of the ZMM proteins and is recruited to the D-loops to promote the formation of SEI (36,39,46). In WT spermatocytes, the number of SHOC1 foci increases with meiotic recombination progression (115.46 ± 5.25 at early-zygotene stage and 151.06 ± 3.97 at late-zygotene stage) and decreases in early-pachynema (102.63 ± 3.27; Figure 4A and Supplementary Figure S6A). In FLIP-deleted spermatocytes, the number of SHOC1 foci (121.10 ± 5.28) is comparable with WT at early-zygotene stage and decreases at zygotene-like stage (104.71 ± 3.16; Figure 4A and Supplementary Figure S6A). TEX11 is another ZMM protein that is recruited to SHOC1-marked SEI and dHJ (47). The overall pattern of TEX11 foci is similar to SHOC1 in WT and FLIP-null spermatocytes (Figure 4B and Supplementary Figure S6B).

Noting that RAD51 and DMC1 are persistent in zygotene-like FLIP-null spermatocytes, a group of recombination intermediates might be positive for both RAD51/DMC1 and SHOC1/TEX11. We set out to analyze the co-localization of RAD51/DMC1 proteins with ZMM proteins. To achieve this, the host origin of the antibodies should be optimized and thus we co-stained SHOC1 (an antibody raised in rabbit)/DMC1 (an antibody raised in mouse)/SYCP3 (an antibody raised in rat) and TEX11 (an antibody raised in goat)/RAD51 (an antibody raised in rabbit)/SYCP3 in spermatocytes derived from WT and *Flip^F/–^;Stra8-Cre* testes. Intriguingly, the co-localizations of SHOC1 with DMC1 and TEX11 with RAD51 are commonly found in FLIP-deleted spermatocytes (Figure 4C–F). There are more foci double positive for SHOC1 and DMC1 in FLIP-null zygonema-like spermatocytes (99.27 ± 8.80) than in WT late-zygotene (44.87 ± 3.67) and early-pachytene (21.20 ± 3.87) spermatocytes (Figure 4D). Similarly, the number of TEX11 and RAD51 double positive foci in FLIP-null zygonema-like spermatocytes is significantly higher than in WT late-zygotene and early-pachytene spermatocytes (Figure 4F).

**Figure 5.**
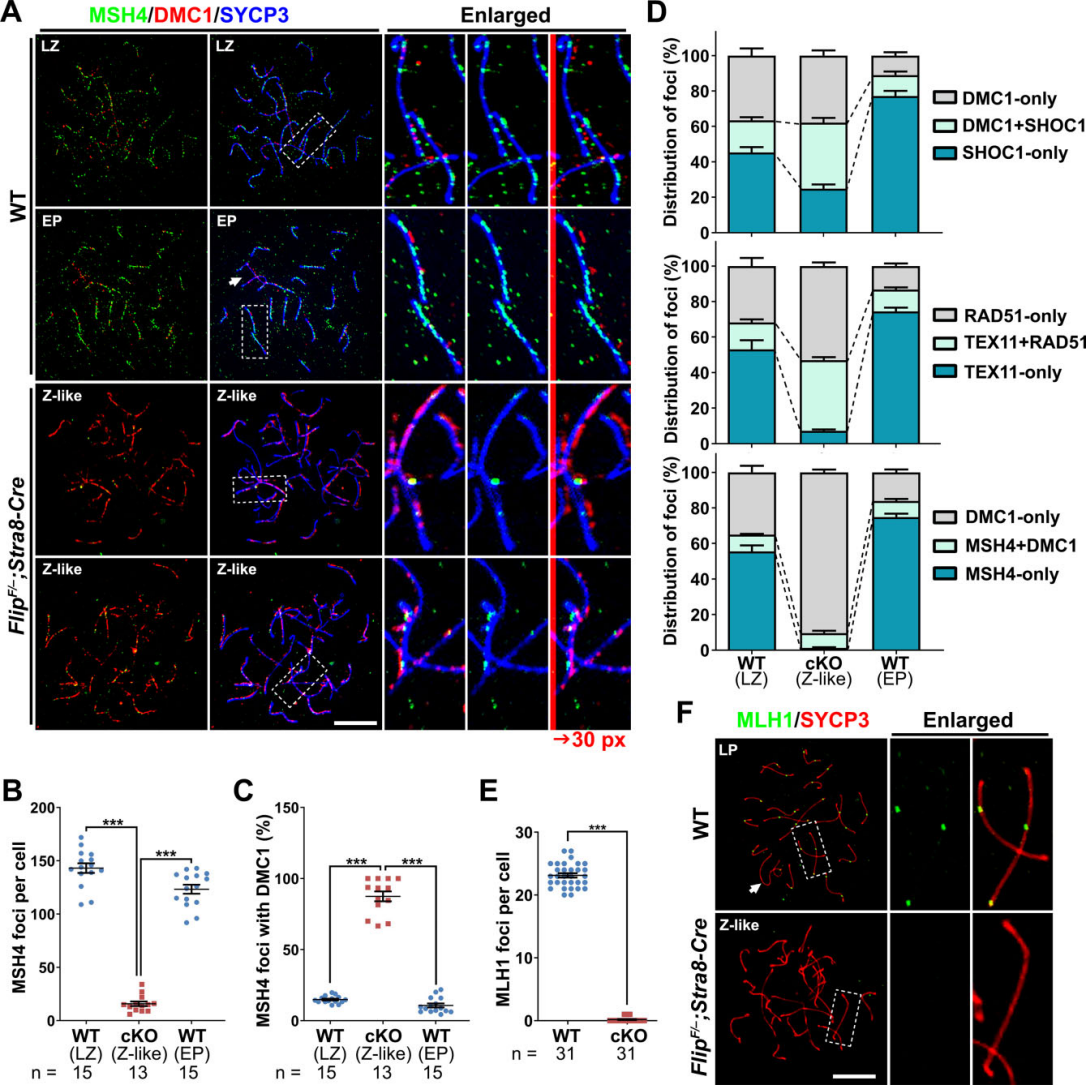
Impaired meiotic homologous recombination in spermatocytes lacking FLIP. (**A**) Triple staining of MSH4 (green), DMC1 (red) and SYCP3 (blue) on nuclear surface spreads prepared with WT and *Flip^F/–^;Stra8-Cre* spermatocytes. The regions within dashed lines are enlarged on the right, and the red signals are further shifted rightwards for 30 pixels to present green-red co-localization. Scale bar, 10 μm. (B, C) Quantification of the MSH4-only foci (**B**) and the MSH4/DMC1 co-localized foci (**C**) in images derived from (**A**). Error bars indicate S.E.M. *n* indicates the numbers of cells analyzed. ***P < 0.001 (two-tailed Student's *t* tests). (**D**) The kinetics of the transitions from RAD51/DMC1-coated filaments to ZMM proteins-marked middle recombination intermediates. Quantification data were collected from immunofluorescent co-staining images of DMC1/SHOC1, RAD51/TEX11 and DMC1/MSH4. The percentages of indicated type of foci in total foci labelled on chromosomes in co-staining analysis were calculated. Error bars indicate S.E.M. (E, F) IF staining (**F**) and the quantification (**E**) of the MLH1 foci (green) contra-stained with SYCP3 (red) on nuclear surface spreads prepared from WT and *Flip^F/–^;Stra8-Cre* testes. The regions within dashed lines are enlarged on the right. Scale bar, 10 μm. Error bars indicate S.E.M. *n* indicates the numbers of cells analyzed. ***P < 0.001 (two-tailed Student's *t* test).

DMC1 were further co-stained with a third ZMM protein, MSH4, which functions downstream of SHOC1 and TEX11 (48). Fewer MSH4 foci were observed in zygotene-like FLIP-null spermatocytes, compared to that in WT late-zygonema and early-pachynema (Figure 5A–C). Moreover, among these rare MSH4 foci in FLIP-deleted spermatocytes at the zygotene-like stage (15.85 ± 2.16 per cell), 87% of them are also DMC1-positive (Figure 5, A and C). These co-localizations are unusual, because in WT spermatocytes the co-localizations of SHOC1/DMC1, TEX11/RAD51 and MSH4/DMC1 are transient, suggesting that FLIP-deletion in spermatocytes impairs the removal of RAD51 and DMC1 from recombination intermediates, which in turn impairs meiotic recombination.

The dynamics of DMC1 versus SHOC1, RAD51 versus TEX11 and DMC1 versus MSH4 are also presented as Figure 5D. In WT spermatocytes, the percentages of DMC1-only and RAD51-only foci decrease during the late-zygotene to early-pachytene transition, while the percentages of SHOC1-only, TEX11-only and MSH4-only foci increase, indicating successful meiotic recombination (Figure 5D). The frequencies of co-localization are below 20% for these protein pairs at late-zygotene and early-pachytene stages. However, in zygotene-like FLIP-null spermatocytes, the co-localization frequencies are 37% and 42% for DMC1/SHOC1 and RAD51/TEX11 pairs, respectively. As a consequence, MLH1 foci, a marker of late recombination intermediates, are almost missing in FLIP-null spermatocytes (Figure 5E, F).

### FLIP forms a complex with FIGNL1

**Figure 6.**
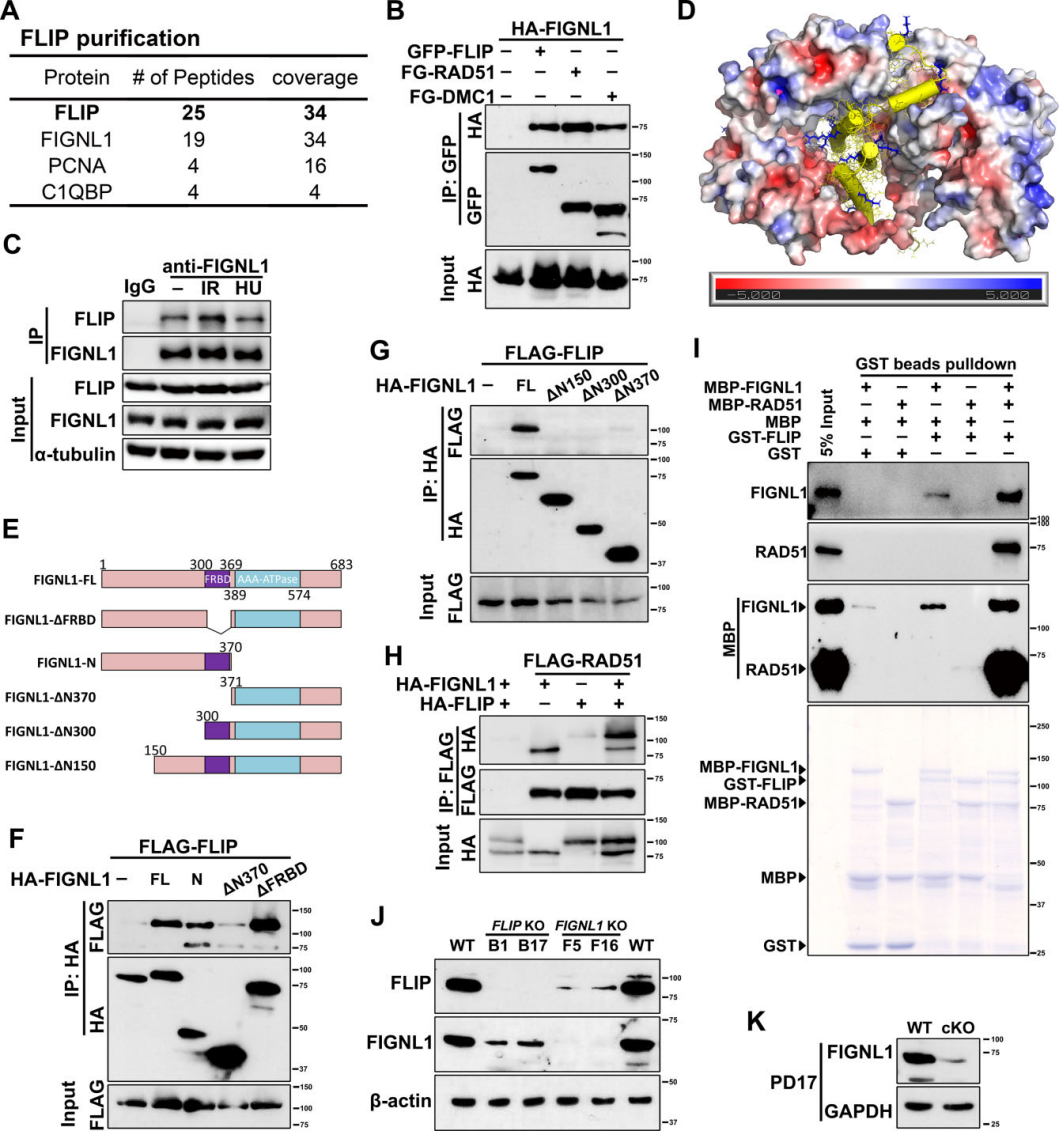
FLIP interacts with the N-terminus of FIGNL1. (**A**) List of proteins identified by TAP and mass spectrometry. Bait proteins are indicated in bold letters. The number of protein peptides and coverages are indicated. (**B**) Immunoprecipitation experiments showing the interaction of FLIP with FIGNL1. RAD51 and DMC1 serve as positive controls. The size of protein markers is indicated. FG, FLAG-EGFP. (**C**) Endogenous FLIP and FIGNL1 form a complex *in vivo*. HEK293T cells were mock treated or treated with 10 Gy or 4 mM HU for 3 h. Whole-cell lysates were immunoprecipitated with either anti-FIGNL1 antibodies or control IgG, followed by immunoblotting with anti-FLIP, and anti-FIGNL1 antibodies. (**D**) Protein structure prediction of the FLIP-FIGNL1 complex showing the interaction between FLIP and the N-terminus 150 amino acids of FIGNL1 (yellow cartoon). FLIP is visualized using electrostatic potential surfaces which are color-coded based on their surface potential (in units of kT/e). Red indicates negatively charged surface, while blue indicates positively charged surface. (**E–G**) Domain mapping of FIGNL1 showing the interaction pattern between FLIP and FIGNL1. FRBD, FIGNL1-RAD51 binding domain. (**H**) IP experiments showing that RAD51 interacts with FLIP via FIGNL1. (**I**) *in vitro* GST pulldown assay showing the complex formation of FLIP, FIGNL1 and RAD51. Immunoblots of FIGNL1, RAD51 and MBP and Coomassie blue staining are provided. (**J**) Western blotting showing the protein levels of FLIP and FIGNL1 in WT, FLIP-deleted and FIGNL1-deleted U2OS cell lines. β-actin serves as loading control. (**K**) Western blotting showing the protein level of FIGNL1 in WT and FLIP-deleted testes at PD17. GAPDH serves as loading control.

To elucidate the mechanism by which FLIP regulates RAD51/DMC1 dissociation, we generated a human HEK293T-derived cell line that stably expresses a S-protein-Flag-streptavidin (SFB)-tagged FLIP for the identification of potential FLIP-interacting proteins. Following a tandem affinity purification (TAP) scheme, proteins associated with FLIP were identified through mass spectrometry analysis. Among the identified proteins, FIGNL1, a known participant in HR repair, was found to be a major binding partner of FLIP (Figure 6A). Consistently, FLIP was found in the anti-FIGNL1 immunoprecipitant (Supplementary Figure S7A). Co-immunoprecipitation experiments confirmed an interaction between FLIP and FIGNL1 (Figure 6B, C). Together with previous findings (18,24), our results suggest that FLIP and FIGNL1 indeed associated with each other *in vivo*. Interestingly, the formation of the FLIP-FIGNL1 complex was found to be independent of DNA or DNA damage (Figure 6C).

In a previous yeast-two-hybrid (Y2H) experiment, both the N-terminal region (aa. 1–290, corresponds to aa. 1–300 in mouse) and the FRBD domain (aa. 291–360, corresponds to aa. 300–369 in mouse) of human FIGNL1 could bind human FLIP (24). To provide molecular detail of the interactions between FLIP and FIGNL1, we used AlphaFold2 multimer to predict the structure of the mouse FLIP-FIGNL1 complex. Our findings showed that FLIP interacted with the N-terminal region (aa. 1–150) of FIGNL1. Specifically, the N-terminal region (aa. 9–79), which consists of a three-helical bundle rich in positively charged residues, inserts perfectly into the negatively charged groove of FLIP (aa. 429–799). This forms a tightly packed complex as shown in Figure 6D. To validate our prediction and investigate the structural dynamics, we performed a 500ns explicit solvent all-atom molecular dynamics simulation of the core regions of the complex. Our results suggest that the complex is very stable (Supplementary Figure S7B) due to extensive hydrogen bonding interactions and salt bridges between FLIP and the N-terminal of FIGNL1 (Supplementary Movie 1). To further delineate the binding interface between FLIP and FIGNL1, we utilized FLAG-tagged wild-type FLIP along with a series of overlapping FLIP truncations and deletion mutants spanning its entire coding sequence (Supplementary Figure S7C-D). Co-immunoprecipitation experiments revealed that FLIP associated with FIGNL1 through its internal region (aa. 351–780). Conversely, using a series of overlapping FIGNL1 truncations and deletion mutants spanning its entire coding sequence, we mapped the FLIP-binding region to residues 1–150 of FIGNL1 (Figure 6E–G). The FRBD domain is not required for the FLIP-FIGNL1 interaction, since FIGNL1-ΔN150 and FIGNL1-ΔN300 truncations containing an intact FRBD domain fail to interact with FLIP (Figure 6G). Compared to the previous Y2H results, we narrowed down the interaction interface of the FLIP-FIGNL1 complex (24).

FIGNL1 is known to bind RAD51 via its FRBD domain and promotes RAD51 dissociation from DNA (18,20). RAD51, together with FLIP, is found in the anti-FIGNL1 immunoprecipitant (Supplementary Figure S7A). FLIP is co-immunoprecipitated by RAD51 and its paralogs, DMC1 and SWSAP1 (Supplementary Figure S7E-F). However, this RAD51-FLIP interaction is strong only in the presence of overexpressed FIGNL1 and decreases to a basal level in the absence of exogenous FIGNL1 (Figure 6H), indicating that FLIP interacts with RAD51 via FIGNL1. To evaluate the direct interaction pattern of the three proteins, we performed *in vitro* GST pulldown assay with the proteins purified in *E. coli*. As shown in Figure 6I, FLIP could interact with RAD51 only in the presence of FIGNL1. The interaction pattern is illustrated in Supplementary Figure S7F.

Moreover, the FLIP-FIGNL1 interaction is essential for the stabilization of both proteins. We have constructed U2OS cell lines null for *FLIP* or *FIGNL1* to analyze the functions of FLIP-FIGNL1 complex in somatic cells (Supplementary Figure S8). In *FLIP* or *FIGNL1* KO U2OS cells, the knockout effects are confirmed by Western blot and interestingly, knockout of *FLIP* decreases the protein level of FIGNL1, and *vice versa* (Figure 6J). In mouse *Flip^F/–^;Stra8-Cre* testes, the protein level of endogenous FIGNL1 is decreased (Figure 6K and Supplementary Figure S9). In WT spermatocytes at the zygotene stage, FIGNL1 forms foci in the nucleus, while in FLIP-null spermatocytes at the zygotene-like stage, less FIGNL1 foci are detected in the nucleus (Supplementary Figure S9).

### The FLIP-FIGNL1 complex regulates RAD51 dissociation to promote somatic recombination

**Figure 7.**
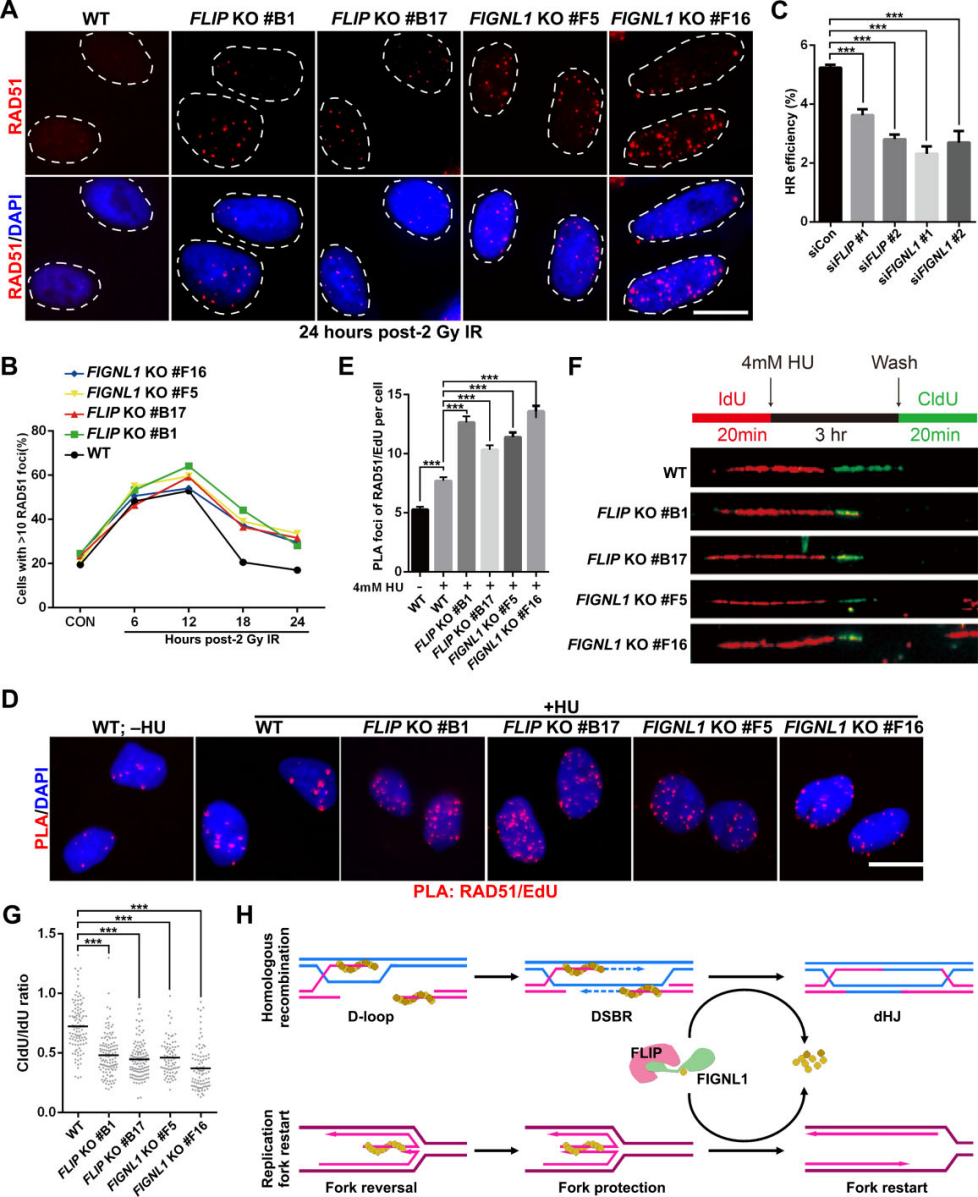
Knockout of FLIP or FIGNL1 impairs RAD51 removal during somatic HR and replication fork restart. (**A**) Immunofluorescent staining of RAD51 in cells at 24 h post 2 Gy IR. Representative RAD51 foci and DAPI-stained nuclei are shown. Scale bar, 10 μm. (**B**) Quantification of RAD51 foci dynamics in WT, *FLIP* KO and *FIGNL1* KO U2OS cells. Data represent mean of three independent experiments. Over 100 cells were counted in each experiment. (**C**) Depletion of FLIP or FIGNL1 impairs HR. U2OS DR-GFP cells transfected with the indicated siRNAs were electroporated with an I-SceI expression plasmid. 48 h after electroporation, cells were harvested and assayed for GFP expression by FACS analysis. Data represent mean ± SEM of three independent experiments. (**D**) Representative images of PLA foci (red). Wildtype, *FLIP* or *FIGNL1* KO U2OS cells were pulse-labeled with 10 μM EdU for 15 min, left untreated or treated with 4 mM HU for 3 h, and then subjected to PLA with anti-RAD51 and anti-biotin (labeling of EdU) antibodies. Scale bar, 10 μm. (**E**) Quantification of PLA foci number per focus positive cell. Data are means ± SEM of three independent experiments. At least 100 cells were counted in each individual experiment. *P < 0.05; ***P < 0.001 (one-way ANOVA tests). (**F**) Top: schematic of the DNA fiber experiment. Bottom: representative IdU and CldU replication tracks in indicated U2OS cells. (**G**) DNA fiber assay following treatment as depicted in (F). Dot plot of CldU to IdU track length ratios for individual replication forks. Data are representative of at least three independent experiments. ***P < 0.001 (one-way ANOVA tests). (**H**) Schematic diagram showing a proposed model of the FLIP-FIGNL1 complex in dismantling RAD51- and/or DMC1-DNA complex during HR and replication fork restart. FIGNL1 interacts with FLIP via its N-terminal domain and forms a FLIP-FIGNL1 heterodimer, which further regulates RAD51/DMC1 dissociation via the FRBD domain of FIGNL1. FLIP is required for both foci formation and protein stability of FIGNL1, and thus deletion of FLIP leads to meiotic recombination arrest at a zygotene-like stage, while deletion of FLIP or FIGNL1 in somatic cells leads to defects in replication fork restart.

The aforementioned results led us to speculate that the FLIP-FIGNL1 complex may also contribute to the dissolution of DNA damage-induced RAD51 foci during the late stage of HR in somatic cells. To test this hypothesis, we analyzed the kinetics of ionizing radiation (IR)-induced RAD51 foci formation in control and in FLIP- or FIGNL1-depleted U2OS cells. As shown in Figure 7A, B and Supplementary Figure S10A, B, in response to IR, RAD51 foci formation increased to approximately 45% at 6 h, peaked at 12 h (around 55%), and then gradually declined in control cells. However, IR-induced RAD51 foci consistently resolved with much slower kinetics in the absence of FLIP or FIGNL1 (Figure 7A, B and Supplementary Figure S10A). Furthermore, knockdown of either FLIP or FIGNL1 resulted in a significant reduction in HR (Figure 7C and Supplementary Figure S10B-C). These results suggest that the FLIP-FIGNL1 complex is critical for RAD51 dissociation and somatic HR.

### The FLIP-FIGNL1 complex regulates RAD51 dissociation to promote replication fork restart

Upon replication stress, RAD51 is recruited to stalled replication forks and binds to first ssDNA and later dsDNA to protect them (49). However, after release from replication stress, the stalled replication forks restart. To investigate whether the FLIP-FIGNL1 complex regulates RAD51 dissociation during replication fork restart and whether RAD51 removal is required for this process, we first performed an *in situ* proximity ligation assay (PLA) to examine the accumulation of RAD51 at stalled forks (Supplementary Figure S10D). As shown in Figure 7D, E, cells treated with HU displayed a dramatic increase in the number of RAD51/EdU PLA foci. Interestingly, RAD51 accumulated to supra-physiological levels at stalled forks in FLIP- and FIGNL1-depleted cells (Figure 7D), indicating that the FLIP-FIGNL1 complex is also required for RAD51 dissociation at stalled replication forks.

We next determined whether the defects in RAD51 dissociation observed in FLIP- or FIGNL1-depleted cells could affect replication fork restart upon cell recovery from replication stress. To assess this, we conducted the DNA fiber assay. In this assay, cells were first pulse-labeled with iododeoxyuridine (IdU), followed by incubation with HU to stall replication forks, and then released into media containing chlorodeoxyuridine (CldU). As shown in Figure 7F, G, deletion of FLIP or FIGNL1 resulted in a significant reduction in the ratio of CldU to IdU track lengths, indicating that inactivation of FLIP or FIGNL1 impairs the restart of stalled forks.

## DISCUSSION

In this study, by engaging *Flip* knockout mice as well as *FLIP* and *FIGNL1* knockout cell lines, we report that the FLIP-FIGNL1 complex is required for the dissociation of RAD51 and DMC1 from joint DNA molecules in meiosis and DNA replication (Figure 7H). First, RAD51 and/or DMC1 foci are stabilized in meiocytes null for FLIP as well as in *FLIP*- and *FIGNL1*-deleted U2OS cells, suggesting a regulatory role of FLIP-FIGNL1 in RAD51/DMC1 dissociation. Second, the FLIP-FIGNL1 complex regulates RAD51/DMC1 dynamics at the post-assembly level. Namely, FLIP-FIGNL1 is not involved in the process of RAD51 loading to ssDNA, but functions in RAD51 dissociation after D-loop formation, which is clearly demonstrated by meiotic HR progression. Third, the direct interaction with FLIP via N-terminal domain is essential for FIGNL1 protein stabilization and foci formation, and thus activity of the FLIP-FIGNL1 heterodimer, to dismantle RAD51–DNA complex.

This mechanism of FLIP-FIGNL1 complex in destabilizing RAD51 and/or DMC1 is conserved not only in different cellular processes, but also in different species. In mice, knockout of *Flip* leads to embryonic lethality, which is consistent with the phenotyping results in IMPC (International Mouse Phenotype Consortium, https://www.mousephenotype.org/data/genes/MGI:3590554). Around 25% deciduae from *Flip^+/–^* to *Flip^+/–^* breeding are found degenerated at E10.5 or E13.5 (Supplementary Figure S1). Degenerated deciduae at E10.5 have the structure of placentas and sometimes remaining gastrulating embryos, suggesting that FLIP-null embryos could develop beyond E6.5, a stage when epiblast, extraembryonic ectoderm and visceral endoderm differentiate to form embryo and extra-embryonic structures (50). The embryonic lethal phenotype restricts further analyses of FLIP in embryogenesis, but manifests its function in HR or DNA replication. The embryonic lethality is observed in mutations of many HR-related proteins, such as *Rad51*, *Blm* and *Xrcc2* (51–53). Therefore, we used *Stra8-Cre* to delete FLIP specifically in meiocytes in testes or ovaries and concluded the indispensable role of FLIP-FIGNL1 complex in meiosis. Increases in RAD51 and/or DMC1 foci are also seen in the germline of worms and plants carrying mutations of FLIP or FIGNL1 homologues (21–24). Moreover, in human cell lines, the FLIP-FIGNL1 complex is critical for RAD51 dissociation from the recombination intermediates during somatic HR and the stalled replication forks during DNA replication, suggesting that the mechanism of FLIP-FIGNL1 complex regulated RAD51 dissociation exists in diverse cellular processes. Knockout of C1ORF112 sensitizes somatic cells to the ICL-inducing compound, mitomycin C, resulting in accumulated DNA damage (27). Tissue-specific Cre might be applied to clarify cell type-specific physiological functions of FLIP and FIGNL1 in different organs in future.

The plant FLIP-FIGL1 complex is believed to be a negative regulator of RAD51 assembly on ssDNA. Different from in mammalian germ cells that FLIP-deletion disrupts CO formation, increased COs are found as the result of increased RAD51/DMC1 in *A. thaliana* (24). BRCA2 (breast cancer 2, early onset) is one of the main mediators of RAD51 that activates RAD51 nucleofilament formation on ssDNA in HR and on replication forks (54,55). Mutation of *figl1* in the background of *brca2* mutation restores RAD51 and/or DMC1 foci formation in both germ cells and somatic cells (25). However, comparable numbers of RAD51/DMC1 foci are detected in FLIP-null spermatocytes at leptotene and early-zygotene stages (Figure 4), as well as in *FLIP*- or *FIGNL1*-deleted somatic cells upon DNA damage (Figure 7). Similarly, in FIGNL1-depleted somatic cells, DNA damage induces similar numbers of RAD51 foci (18). These results suggest normal levels of RAD51 and/or DMC1 recruitment in mammalian cells null for FLIP or FIGNL1, and thus, a post-assembly role of the FLIP-FIGNL1 complex in regulating RAD51/DMC1. It is known that the RAD51-DNA complexes are negatively regulated by many HR-related proteins, such as BLM, RECQL5, FBH1, FANCJ and PARI, *etc* (56–60). However, these proteins function mainly in limiting formation of joint molecules on undesired sites and thereby ensure genome stability. On the other hand, increased RAD51 and DMC1 foci in FLIP-null meiocytes could also be attributed to defects in D-loop stabilization, leaving RAD51/DMC1-coated 3′-ssDNA overhangs, as seen in *Shoc1* or *Tex11*-knockout meiocytes (36,47). However, the retention of RAD51/DMC1 foci is found on SHOC1- and TEX11-positive recombination intermediates, suggesting a role of the FLIP-FIGNL1 complex in regulating RAD51/DMC1 removal at the step of RAD51/DMC1-dsDNA complexes (D-loop, SEI, and dHJ), instead of RAD51/DMC1–ssDNA complexes.

Through structure prediction and molecular docking, the N-terminal 150 aa on FIGNL1 is identified to interact with FLIP, which is further confirmed by domain mapping and Co-IP. The complex formation is essential for the stabilization of both proteins and foci formation of FIGNL1, therefore could be a prerequisite for their molecular functions. Interestingly, FLIP is shown to be associated to stalled replication forks by iPOND (61). FIGNL1 N-terminal deletions have been previously studied in somatic HR via cellular and biochemical analyses. N-terminal 120 aa deletion of human FIGNL1 (corresponds to mouse FIGNL1-ΔN127) is reported to disrupt its foci formation on DSBs (18). In another study, a FIGNL1-ΔN284 (corresponds to mouse FIGNL1-ΔN293) truncation is used to reveal its function in dismantling RAD51 filaments *in vitro* (20). However, FIGNL1-ΔN284 exhibits a relatively low activity in disrupting RAD51-DNA complexes, when compared to other RAD51 negative-regulators (57,62). Taken together, we infer that the association of FLIP and FIGNL1 guarantees their individual protein stability as well as activity, and promotes FIGNL1 to dismantle RAD51 and DMC1 from recombination intermediates and stalled replication forks.

## Supplementary Material

gkad596_Supplemental_Files

## Data Availability

All data needed to evaluate the conclusions in the paper are present in the paper and/or the Supplementary Materials. Additional data related to this paper may be requested from the authors.
