# Supplementary material for: The FLIP-FIGNL1 complex regulates the dissociation of RAD51/DMC1 in homologous recombination and replication fork restart
Source: Nucleic Acids Res. 2023 Jul 13;51(16):8606–22. doi: 10.1093/nar/gkad596 (PMC10484675; doi:10.1093/nar/gkad596)
Supplement: gkad596_Supplemental_Files [file gkad596_supplemental_files.zip › FLIP_4_Sup.pdf]

Supplementary information

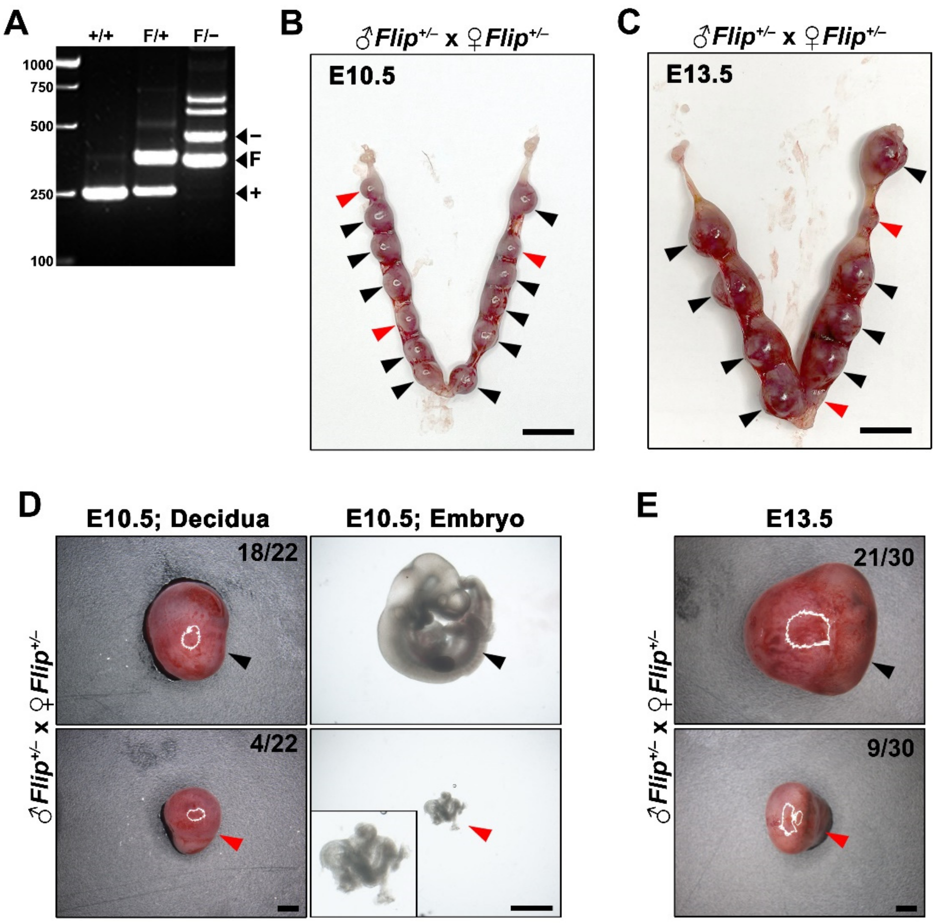

Supplementary Figure S1. Deletion of FLIP leads to embryonic lethality. (**A**) Representative genotyping image showing the bands for Wildtype allele (+), floxed allele (F) and null allele (–) of *Flip*. (**B-C**) Images of the ovaries and uteruses at embryonic day 10.5 (E10.5) and E13.5. *Flip*$^{+/-}$ males were crossed to *Flip*$^{+/-}$ females. Vaginal plugs were checked in the next morning, which corresponded to E0.5. Black and red arrowheads indicate normal and degenerated embryos, respectively. Scale bars, 1 cm. (**D**) Deciduae were dissected from the uterus in (**B**) and embryos were further dissected from deciduae. 18/22 and 4/22 indicate the number of normal (18) and degenerated deciduae (4) out of total number of deciduae (22). Scale bars, 1 mm. (**E**) Deciduae were dissected from the uterus in (**C**). 21/30 and 9/30 indicate the number of normal (21) and degenerated deciduae (9) out of total number of deciduae (30). Scale bar, 1 mm.

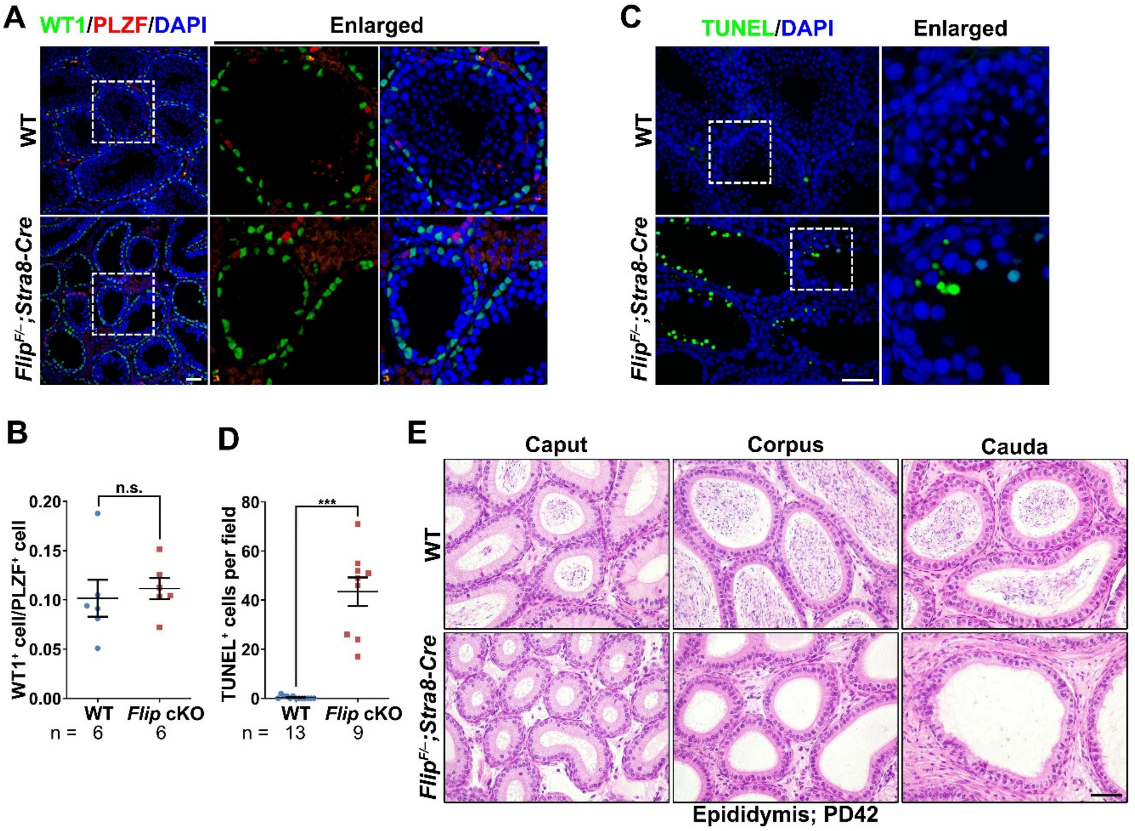

Supplementary Figure S2. The processes of spermatogenesis are affected by FLIP-deletion. (**A**) IF staining of WT1 (green) and PLZF (red) in WT and *Flip*$^{F/-}$*;Stra8-Cre* testes sections at PD42. The regions with dashed lines are enlarged on the right. Scale bar, 500 µm. (**B**) The ratios of WT1-positive cells to PLZF-positive cell are quantified in WT and *Flip*$^{F/-}$*;Stra8-Cre* testes. Error bars indicate S.E.M. The numbers of sections analyzed (n) are indicated. n.s., $P > 0.05$ (two-tailed Student's *t* tests). (**C-D**) TUNEL (TdT-mediated dUTP Nick-End Labeling) assay showing the apoptotic cells in WT and FLIP-deleted testes. The quantification of TUNEL-positive cells is illustrated in (**D**). Scale bar, 50 µm. Error bars indicate S.E.M. The numbers of sections analyzed (n) are indicated. ***, $P < 0.0001$ (two-tailed Student's *t* tests). (**E**) H&E staining of epididymides derived from WT and *Flip*$^{F/-}$*;Stra8-Cre* males at PD42. Caput, corpus and cauda indicate different parts of epididymis. Scale bar, 50 µm.

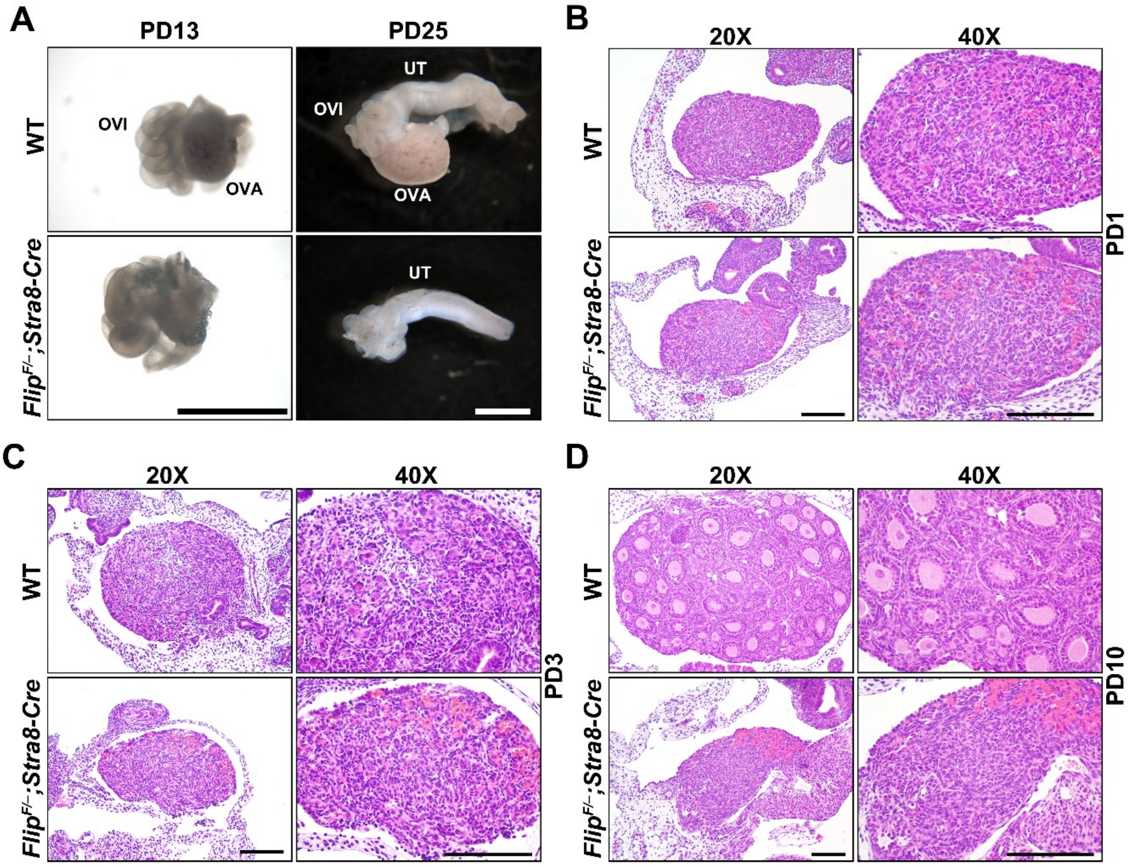

Supplementary Figure S3. ***Flip$^{F/-}$;Stra8-Cre*** **females exhibit the phenotype of primordial ovarian insufficiency (POI).** (**A**) Representative images showing the morphology of ovaries and the surrounding oviducts and uteruses at the indicated ages. OVA, ovary; OVI, oviduct; UT, uturus. Scale bars, 1 mm. (**B-D**) H&E staining of WT and *Flip$^{F/-}$;Stra8-Cre* ovaries at PD1, PD3 and PD10. Scale bars, 100 µm.

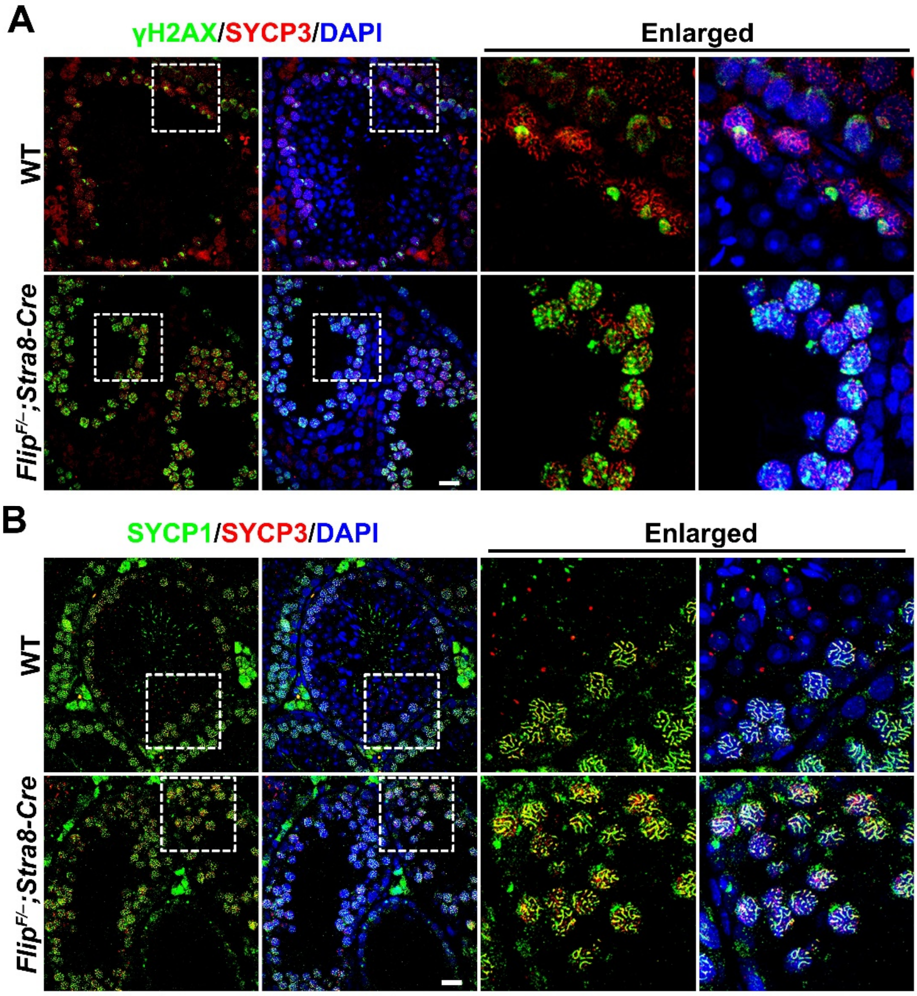

Supplementary Figure S4. Spermatogenesis in FLIP-deleted spermatocytes is arrested at the zygotene-like stage. (**A-B**) Co-staining of γH2AX (**A**, green) or SYCP1 (**B**, green) and SYCP1 (red) in WT and *Flip*$^{F/-}$*;Stra8-Cre* testes at PD42, showing the status of DSB repair and synaptonemal complex formation in spermatocytes. The regions with dashed lines are enlarged on the right. Scale bars, 20 μm.

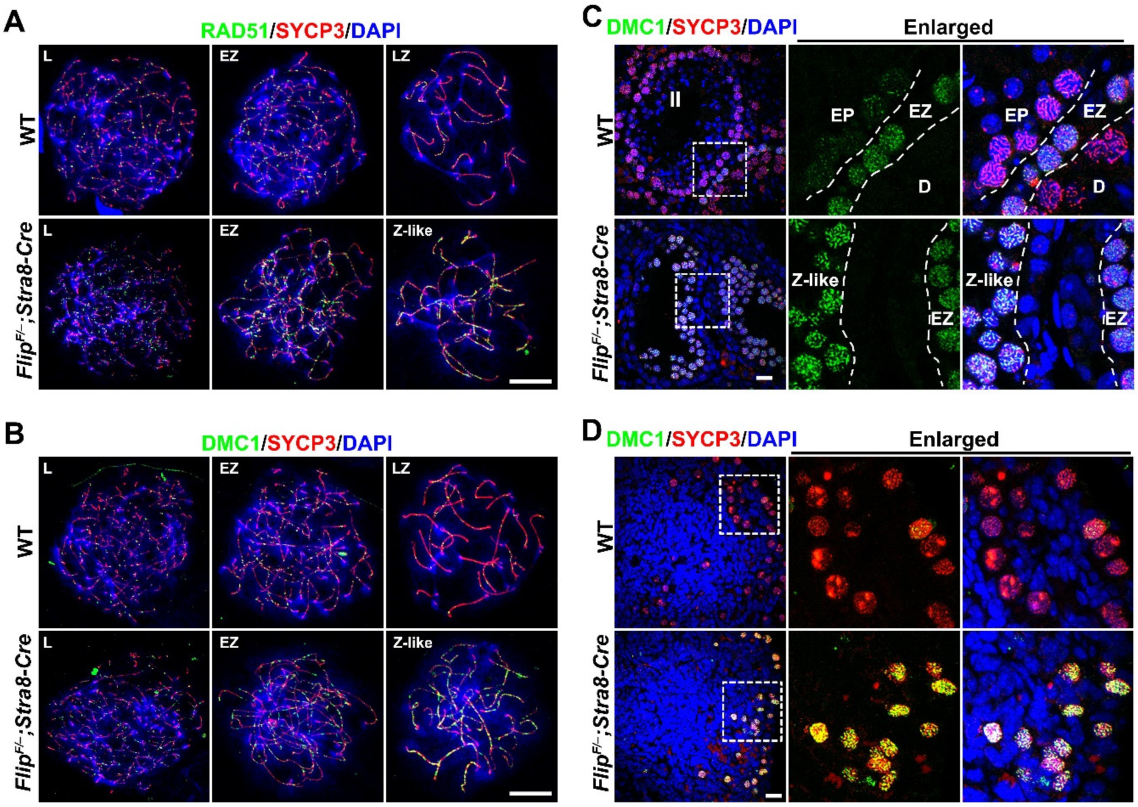

Supplementary Figure S5. Dynamics of RAD51 and DMC1 in WT and FLIP-deleted meiocytes. (**A-B**) RAD51 (**A**) and DMC1 (**B**) were detected on the nuclear surface spreads of WT and *Flip$^{F/–}$;Stra8-Cre* spermatocytes at indicated stages. Scale bars, 10 µm. (**C-D**) Co-staining of DMC1 (green) and SYCP1 (red) on testis sections at PD42 (**C**) and ovary sections at PD1 (**D**). The stages of spermatocytes are indicated. II, a representative seminiferous tubule at stage II. Scale bars, 20 µm.

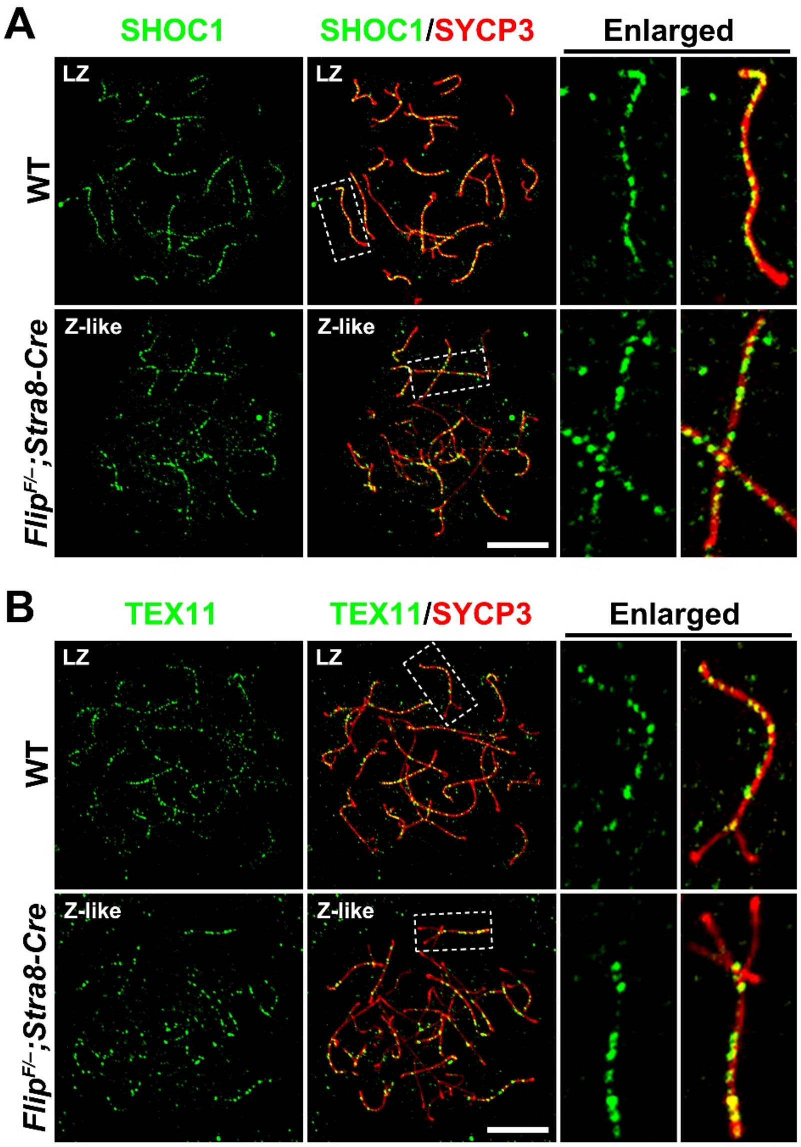

Supplementary Figure S6. Dynamics of ZMM proteins in WT and FLIP-deleted meiocytes. IF staining of SHOC1 (**A**, green) or TEX11 (**B**, green) and SYCP3 (red) on the nuclear surface spreads of WT and *Flip*$^{F/–}$*;Stra8-Cre* spermatocytes at indicated stages. The regions with dashed lines are enlarged on the right. Scale bars, 10 μm.

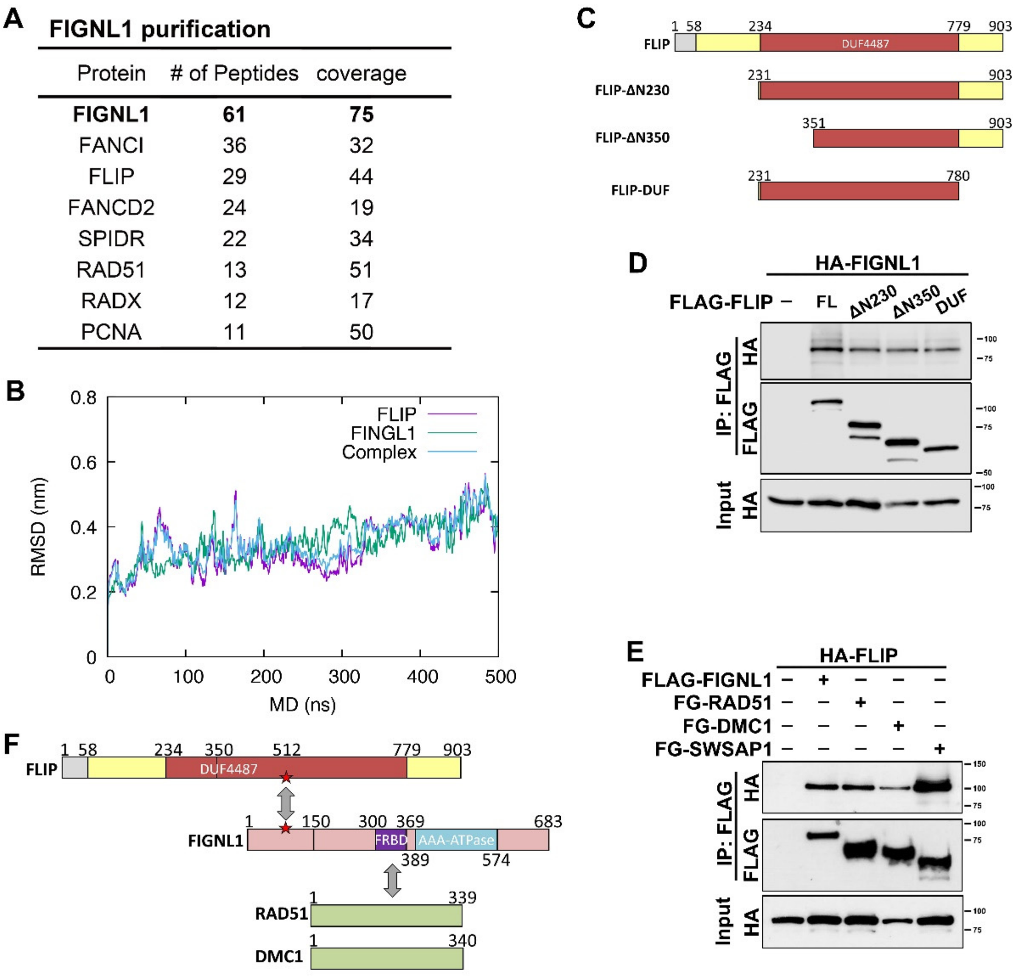

Protein	# of Peptides	coverage
**FIGNL1**	**61**	**75**
FANCI	36	32
FLIP	29	44
FANCD2	24	19
SPIDR	22	34
RAD51	13	51
RADX	12	17
PCNA	11	50

Supplementary Figure S7. Interaction between FLIP and FIGNL1. (**A**) List of proteins identified by TAP and mass spectrometry of anti-FIGNL1 immunoprecipitant. Bait proteins are indicated in bold letters. The number of protein peptides and coverages are indicated. (**B**) A 500ns explicit solvent all-atom molecular dynamics simulation of the core regions of the complex. RMSD, root mean square deviation. (**C-D**) Domaining mapping of FLIP. Schematic diagram (**C**) illustrating the wildtype and four deletions of FLIP (mouse) constructed in the lab to investigate the FIGNL1-binding domain. IP results (**D**) showing the interaction of FIGNL1 with full-length FLIP (FL) and its deletions. (**E**) IP results showing the interaction of FLIP with FIGNL1, RAD51, DMC1 and SWSAP1 via overexpressing cDNAs derived from mice. (**F**) Schematic diagram showing the interaction pattern of FLIP, FIGNL1 and RAD51/DMC1. Numbers indicate the locations of amino acids. FRBD, FIGNL1-RAD51 binding domain.

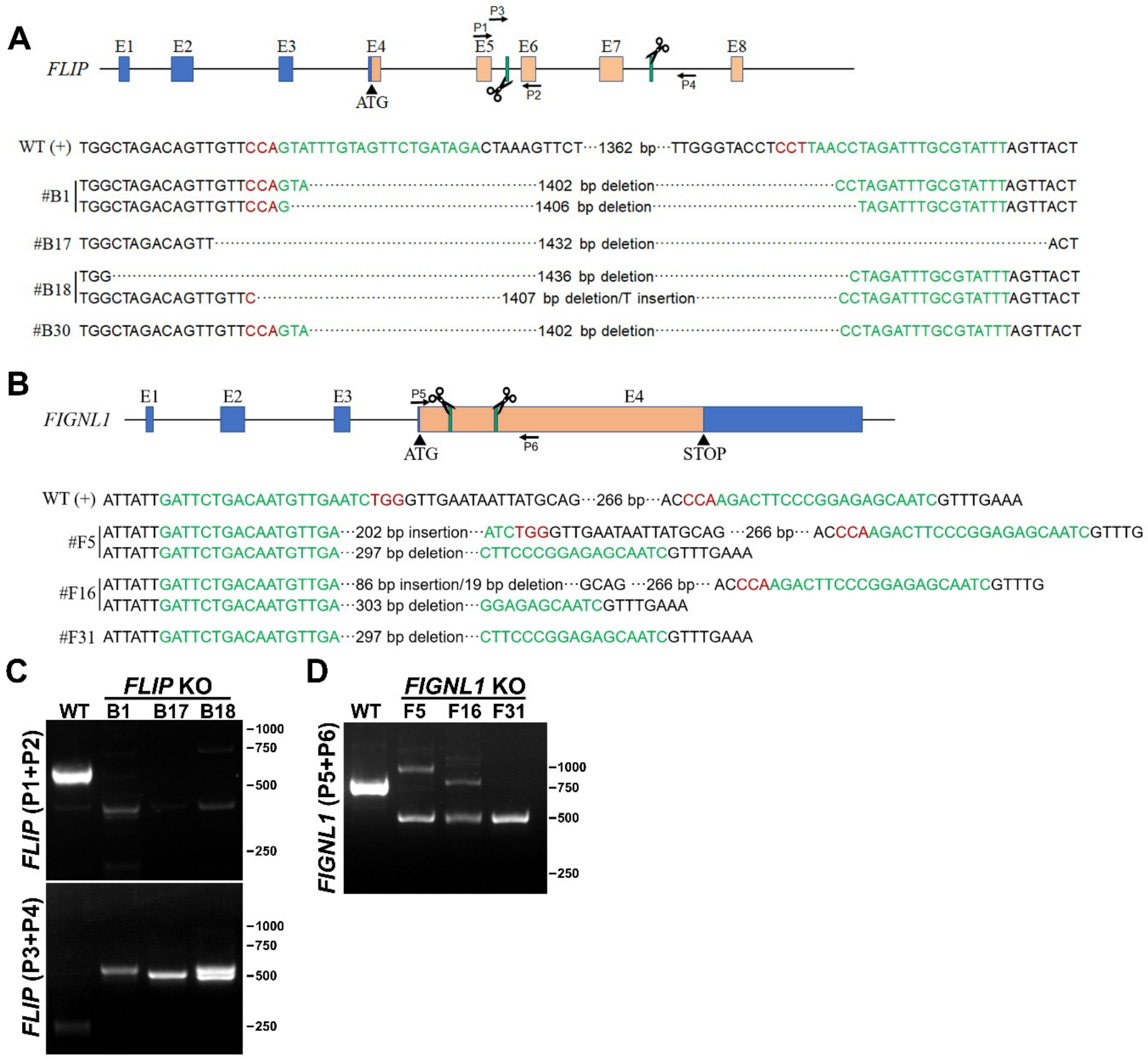

Supplementary Figure S8. Construction of knockout U2OS cells for *FLIP* and *FIGNL1*. (**A-B**) Schematic showing the CRISPR/Cas9 strategy to generate *FLIP* (**A**) or *FIGNL1* (**B**) knockout (KO) human cell lines. Exons, sites of sgRNA and primers are indicated. The serial numbers such as #B1 and #F5 represent the single colonies verified carrying two *FLIP* or *FIGNL1*-KO alleles. The varied sequences in WT and *FLIP* or *FIGNL1*-KO alleles of these colonies are listed. (**C-D**) Genotyping results of *FLIP* KO and *FIGNL1*-KO U2OS cell lines.

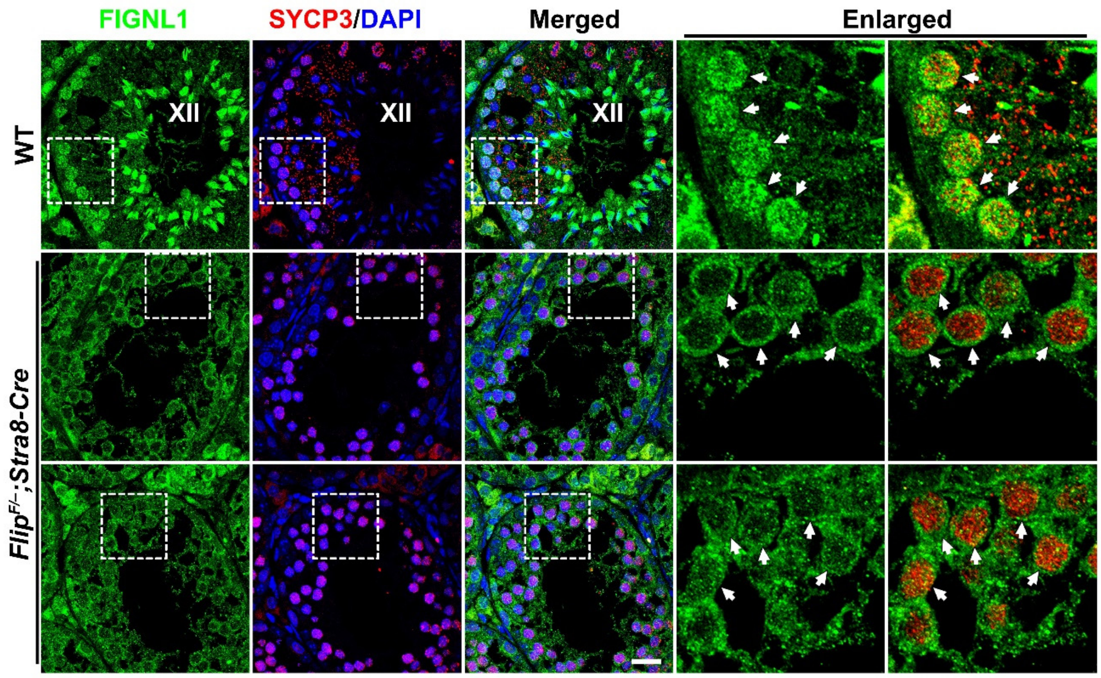

Supplementary Figure S9. Immunofluorescent staining of FIGNL1 on testes sections derived from WT and *Flip*$^{F/–}$*;Stra8-Cre* males. Arrow heads indicate zygotene spermatocytes in WT testes and zygotene-like spermatocytes in *Flip*$^{F/–}$*;Stra8-Cre* testes. Scale bar, 20 μm.

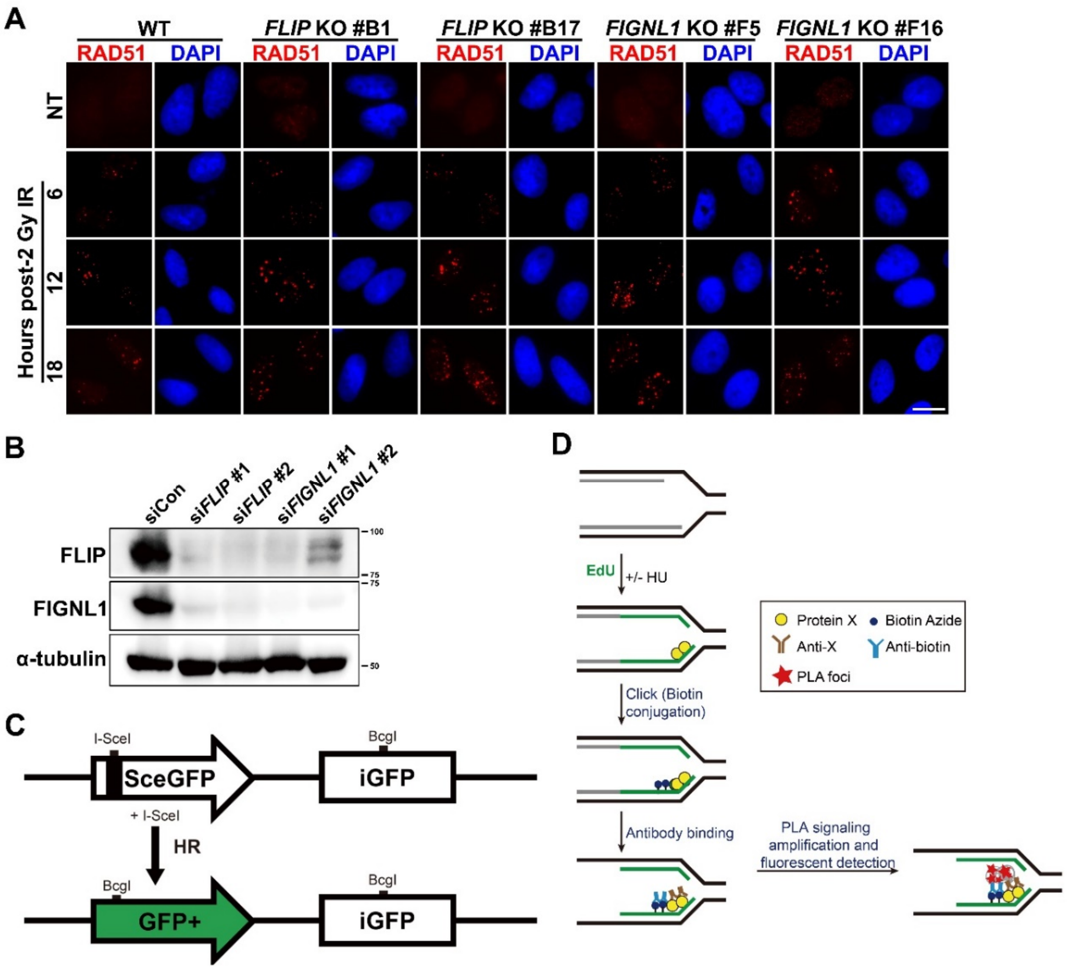

Supplementary Figure S10 . Knockout of FLIP or FIGNL1 impairs RAD51 removal in cell lines. (A) Cells were exposed to 2 Gy IR and then allowed to recover for the indicated time periods before being processed for immunofluorescence using antibodies against RAD51. Representative RAD51 foci and DAPI-stained nuclei are shown. Scale bar, 10 µm. (B) Knockdown efficiency of FLIP and FIGNL1 via indicated siRNAs was confirmed by western blotting. (C) Schematic representation of HR assay. The DR-GFP construct consists of direct repeats of two mutated GFP genes, SceGFP and the truncated iGFP. When a single double-strand DNA break generated by I-Sce1 is repaired via gene conversion with iGFP, the expression of GFP is restored and can be measured by FACS analysis. (D) Schematic of the proximity ligation assay (PLA) utilized to detect the association of proteins with nascent DNA. Wildtype, *FLIP* or *FIGNL1* KO cells were pulse-labeled with 10 µM EdU for 15 min, left untreated or treated with 4 mM HU for 3 h, and then subjected to PLA with anti-RAD51 and anti-biotin antibodies.

Supplementary Movie 1. A 500ns explicit solvent all-atom molecular dynamics simulation of the core regions of FLIP (aa. 9-79) and FIGNL1(aa. 429-799).

Supplementary Table S1. Primer sequences.

**Primer name**	**Genes targeted**	**Application**	**Sequences (5'-3')**
Z491	*Flip*	Genotyping (254 bp/359 bp/463 bp for WT/Floxed/null alleles)	5'-TGGGACCAGATAACTTGAGTAGC-3'
Z492			5'-AGACAAGGTCTCTCTACATAGCCC-3'
Z493			5'-GTTAGGAATATAATCTTTGCTTGCC-3'
Z051	*Stra8-Cre*	Genotyping (702 bp/400 bp for WT/KI allele)	5'-ACTCCAAGCACTGGGCAGAA-3'
Z052			5'-GCCACCATAGCAGCATCAAA-3'
Z053			5'-CGTTTACGTCGCCGTCCAG-3'
P1	*FLIP*	Genotyping (581 bp/513 bp for WT/KO allele)	5'-CAGTCAAGCCAGAGGACTGTC-3'
P2			5'-ATGATGGATTCCTGTGTGGC-3'
P3			5'-AGTCAAATGTATTAGAAAGCAGGAG-3'
P4			5'-TGCATCACATGTAAAATGAGTTATT-3'
P5	*FIGNL1*	Genotyping (800 bp/469 bp for WT/KO allele)	5'-TCTGTGCACCTGAGTGAATGG-3'
P6			5'-AGTATTGGATTGGAAAGTGCATC-3'

Supplementary Table S2. Information for antibodies and agarose beads.

Protein name	Manufacture (catalogue number)	Origin	Applications (working dilution)	Website Link
SYCP1	Abcam (ab15087)	Rabbit	IF (1:200)	https://www.citeab.com/antibodies/771942-ab15087-anti-scp1-antibody
SYCP3	homemade	Rat	IF (1:500)	Immunogen: Full length of mouse SYCP3
FLIP	Abcam (ab121774)	Rabbit	WB (1:1000)	https://www.abcam.cn/c1orf112-antibody-ab121774.html
FLIP	Abnova (PAB21606)	Rabbit	WB (1:1000)	http://www.abnova.com/products/products_detail.asp?catalog_id=PAB21606
GAPDH	Shanghai Genomics (GNI4110-GH)	Mouse	WB (1:3000)	http://www.gnimission.com/resource/article/76
MVH	Abcam (ab13840)	Rabbit	IF (1: 200); IHC (1: 400)	http://www.abcam.com/ddx4--mvh-antibody-ab13840.html
PLZF	Santa Cruz (sc28319)	Mouse	IF (1: 200)	https://www.scbt.com/p/plzf-antibody-d-9/
γH2AX	Cell Signaling (9718S)	Rabbit	IF (1: 400)	https://www.cellsignal.com/products/primary-antibodies/phospho-histone-h2a-x-ser139-20e3-rabbit-mab/9718?N=4294956287&Ntt=h2a.x&fromPage=plp
HORMAD1	Abcam (ab155176)	Rabbit	IF (1: 200)	http://www.abcam.com/hormad1-antibody-ab155176.html
RAD51	Abcam (ab176458)	Rabbit	IF (1: 200)	http://www.abcam.com/rad51-antibody-chip-grade-ab176458.html
DMC1	Abcam (ab11054)	Mouse	IF (1: 100)	http://www.abcam.com/dmc1-antibody-2h124-ab11054.html

SHOC1/ MZIP2	homemade	Rabbit	WB (1:500); IF (1:100)	Immunogen: aa474-635 of mouse SHOC1/MZIP2; Immunized by Core facilities, Zhejiang University School of Medicine.
TEX11	R&D systems (AF5627-SP)	Goat	IF (1: 200)	https://www.rndsystems.com/cn/products/mouse-zip4h-tex11-antibody_af5627#product-datasheets
MSH4	Abcam (ab58666)	Rabbit	IF (1: 50)	http://www.abcam.com/msh4-antibody-ab58666.html
MLH1	BD (551092)	Mouse	IF (1: 200)	http://www.bdbiosciences.com/us/applications/research/apoptosis/purified-antibodies/purified-mouse-anti-mlh-1-with-control/p/551092
FLAG	Sigma-aldrich (F3165)	Mouse	WB (1: 3000)	http://www.sigmaaldrich.com/catalog/product/sigma/f3165?lang=en&region=SE
FLAG	CST (14793S)	Rabbit	WB (1: 1000)	https://www.cellsignal.cn/products/primary-antibodies/dykddddk-tag-d6w5b-rabbit-mab-binds-to-same-epitope-as-sigma-s-anti-flag-m2-antibody/14793
HA	Shanghai Genomics (GNI4110-HA-P)	Mouse	WB (1:1000)	http://www.gnimission.com/resource/article/201
HA	CST (3724S)	Rabbit	WB (1: 1000)	https://www.cellsignal.cn/products/primary-antibodies/ha-tag-c29f4-rabbit-mab/3724
GFP	Shanghai Genomics (GNI4110-GP)	Mouse	WB (1:1000)	http://www.gnimission.com/resource/article/88
FIGNL1	Proteintech (17604-1-AP)	Rabbit	WB (1:1000)	https://www.ptgcn.com/products/FIGNL1-Antibody-17604-1-AP.htm

β-actin	Shanghai Genomics (GNI4110-BA)	Mouse	WB (1:1000)	http://www.gnimission.com/resource/article/206
WT1	abcam (ab89901)	Rabbit	IF 1:100	https://www.abcam.cn/products/primary-antibodies/wilms-tumor-protein-antibody-can-r9ihc-56-2-ab89901.html
IdU/BrdU	BD Biosciences (347580)	Mouse	IF 1:500	https://www.bdbiosciences.com/zh-cn/products/reagents/flow-cytometry-reagents/clinical-discovery-research/single-color-antibodies-ruo-gmp/purified-mouse-anti-brdu.347580
CldU/BrdU	abcam (ab6326)	Rat	IF 1:500	https://www.abcam.cn/products/primary-antibodies/brdu-antibody-bu175-icr1-proliferation-marker-ab6326.html
α-tubulin	Abmart M20005S	Mouse	WB 1:3000	http://www.ab-mart.com.cn/page.aspx?node=%2059%20&id=%20983
Biotin	Jackson ImmunoResearch (200-002-211)	Mouse	PLA 1:3000	https://www.jacksonimmuno.com/catalog/products/200-002-211
ANTI-FLAG® M2 Affinity Gel	Sigma (A2220)	Mouse		https://www.sigmaaldrich.cn/CN/en/product/sigma/a2220
Anti-HA Affinity Gel Plus	Shanghai Genomics (GNI4510-HA-P)	Mouse		http://www.gnimission.com/resource/article/202
Anti-GFP	AlpalifeBio™ (KTSM1301)	Alpaca		http://www.ktsm-life.com/product-detail-260.html

Streptavidin Sepharose HP	GE (GE17-5113-01)			https://www.sigmaaldrich.com/HK/zh/product/sigma/ge17511301
S-protein Agarose	Millipore (69704-4)			https://www.merckmillipore.com/CN/zh/product/S-protein-Agarose,EMD_BIO-69704

Supplementary Table S3. siRNA sequences.

**siRNA**	**Sequences (5’-3’)**
si*FLIP* #1	5’- GCCAATACTTGGAAGTTTA -3’
si*FLIP* #2	5’- CAGGATATCTCTACTCAAA -3’
si*FIGNL1* #1	5’- GGATCAAGTTCGACCCATA -3’
si*FIGNL1* #2	5’- GAATGAGATTATGGATCAT -3’
